# The spatial propagation and increasing dominance of *Gilbertiodendron dewevrei* (Fabaceae) in the eastern Congo basin

**DOI:** 10.1371/journal.pone.0275519

**Published:** 2023-02-07

**Authors:** Henry B. Glick, Peter M. Umunay, Jean-Remy Makana, C. Dana Tomlin, Jonathan D. Reuning-Scherer, Timothy G. Gregoire

**Affiliations:** 1 School of the Environment, Yale University, New Haven, CT, United States of America; 2 The Global Environmental Facility (GEF), Washington DC, United States of America; 3 Faculty of Science, University of Kisangani, Kisangani, Democratic Republic of Congo; 4 Weitzman School of Design, University of Pennsylvania, Philadelphia, PA, United States of America; University of Fribourg, SWITZERLAND

## Abstract

Though substantial research has been conducted on possible historical, physiological, and symbiotic mechanisms that permit monodominance to occur within tropical lowland rainforests, less is known about the successional rates at which monodominance exerts itself on surrounding forest structures. Here we extend efforts to evaluate the longitudinal dynamics of *Gilbertiodendron dewevrei*-dominated forest in Central Africa by considering this species’ spatial dynamics. Using three 10-ha censused field plots measured across three time periods, we present the first quantitative estimates of the spatial propagation of *Gilbertiodendron* into adjacent mixed species forest. Using three analytical strategies, we demonstrate that *Gilbertiodendron* is increasing in dominance and that monodominant forest patches are expanding into the surrounding forest at a statistically significant rate. The rates of successional advance vary by patch and direction, but average 0.31 m year^−1^, with speeds greatest in the direction of the prevailing winds. We show that the advancement of *Gilbertiodendron* is significantly slower than documented rates from other forest ecotones across Central Africa. When paired with stress tolerance traits and ectomycorrhizal associations, these findings help to clarify the means by which *Gilbertiodendron dewevrei* gains dominance in otherwise species-diverse regions.

## Introduction

The structural composition and changing dynamics of tropical lowland rainforests have piqued scientific interest for decades [1–6]. Tropical forests often garner attention through their unusually high species diversity, though species monodominance presents an equally engaging phenomenon [7]. Although reduced species diversity owing to species monodominance has historically been considered unusual [4, 8–10], we know that monodominance occurs on all tropical forested continents [9, 11, 12]. Yet, the reasons and mechanisms for monodominance in otherwise species-diverse forest complexes remains controversial and somewhat mysterious [8, 9, 13–15]. Here, we focus on the forests of Central Africa’s Congo River basin and the manner in which *Gilbertiodendron dewevrei*, a long-lived, leguminous climax species in *Caesalpinioideae* [16], exerts its influence on the local landscape.

In the Congo River basin, *Gilbertiodendron dewevrei* (hereafter *Gilbertiodendron*) possesses physiological and life history traits that appear to confer a competitive advantage over local conspecifics [15–17], including over other climax species such as *Julbernadria seretii* and *Cynometra alexandri*. These traits include an ectomycorrhizal association [8, 12, 15, 17], large seeds and mast crops [12, 15, 18], deep tap roots [3, 17], tolerance for deep shade [18], plasticity in light acquisition [17, 19], and recruitment that influences microsite conditions [16, 24, 17] (see also [13] Supplementary material). The interaction between *Gilbertiodendron* and the adjacent species-diverse mixed species forest assemblages is nuanced, and there has been substantial research into how and/or why these forest types differ (e.g., [14–16, 20–24]). *Gilbertiodendron*-dominated forest can generally be regarded as a late successional climax community, and, following the nomenclature of Connell and Lowman [8] and Newbery *et al.* [25], an example of type I monodominance. Type I monodominance is that in which a dominant canopy species is able to maintain dominance beyond a single generation in the absence of large-scale disturbance. Over time, these traits have promoted *Gilbertiodendron*-dominated forest structures from the eastern Democratic Republic of the Congo (DRC), west to Nigeria [14]. Where *Gilbertiodendron* dominates, it can represent 63–90% of canopy specimens in relatively homogeneous blocks of up to several thousand hectares [4, 5, 14, 16, 21, 26]—structural characteristics that are unique among global rainforests [4, 12, 16].

Though *Gilbertiodendron*-dominanted forests have been studied for the better part of a century [2, 14, 16, 20, 26], we still lack a unified understanding of the mechanistic processes by which their monodominance occurs, and a clear understanding of their rates of forest succession. To better understand the manner in which a mixed-species forest composition yields to monodominance, Glick *et al.* [13] examined shifting forest types in the Ituri region of the DRC using a successional trajectory defined by: mixed species forest, in which *Gilbertiodendron* represented <5% of the total living basal area (BA); transitional forest, in which *Gilbertiodendron* represented ≥5% and <50% of the BA; and *Gilbertiodendron*-dominated forest, in which *Gilbertiodendron* represented ≥50% of the BA. Statistically significant results suggest that the forests of the Ituri region have followed a uni-directional successional trajectory leading from mixed species to transitional to *Gilbertiodendron*-dominated forest. Thomas *et al.* [27] also presented findings that considered the transition from mixed species forest to *Gilbertiodendron*-dominated forest. Using two of the three censuses utilized in the present work, these authors found that in the Ituri region of the DRC, the boundary between *Gilbertiodendron*-dominated forest and mixed species forest was moving towards the latter at a rate of 0.34 m per year (per [28]). Our research was motivated by this initial effort.

In the present study, we sought to extend these efforts by focusing on one over-arching question: Is *Gilbertiodendron dewevrei* expanding into the surrounding mixed species forest, effectively converting mixed species assemblages into monodominant forest? We focused on three hypotheses that (a) targeted complementary measures of *Gilbertiodendron*’s propagation and displacement over space, along with the associated displacement of mixed species forest; and that (b) capitalized on unique computational strategies. We hypothesized that:

1. When looking across each field site, the spatial extent of *Gilbertiodendron*-dominated forest patches has increased substantially over time.
2. Relative to patch centroids, *Gilbertiodendron*-dominated forest patch boundaries have migrated outward, reflecting patch expansion across time.
3. The magnitudes and directions of change over time in *Gilbertiodendron* BA intensity (BA/m^2^), reflect both an increase in BA and greater rates of change around the margins of regions with higher BA concentrations.

Late successional monodominance is generally characterized by slow-growing, shade tolerant species with large seeds and poor dispersal [9]. Slow growth and poor seed dispersal would tend to contraindicate widespread success and short-term influence. However, previous research has indicated that *Gilbertiodendron* can be a key determinant in forest structure [15, 18, 21] with increasing success over relatively short time periods [13]. Here, we take a more direct approach to (a) examine whether *Gilbertiodendron* is advancing into surrounding forest structures, and to (b) quantify the rate of that movement.

## Materials and methods

All data management, analysis, and visualization tasks were performed using R v. 3.5.3 (R Core Team, 2019). Specific packages and workflows are described below, and are available in Supporting Information S1 Data.

### Field plots and data structures

**Fig 1:**
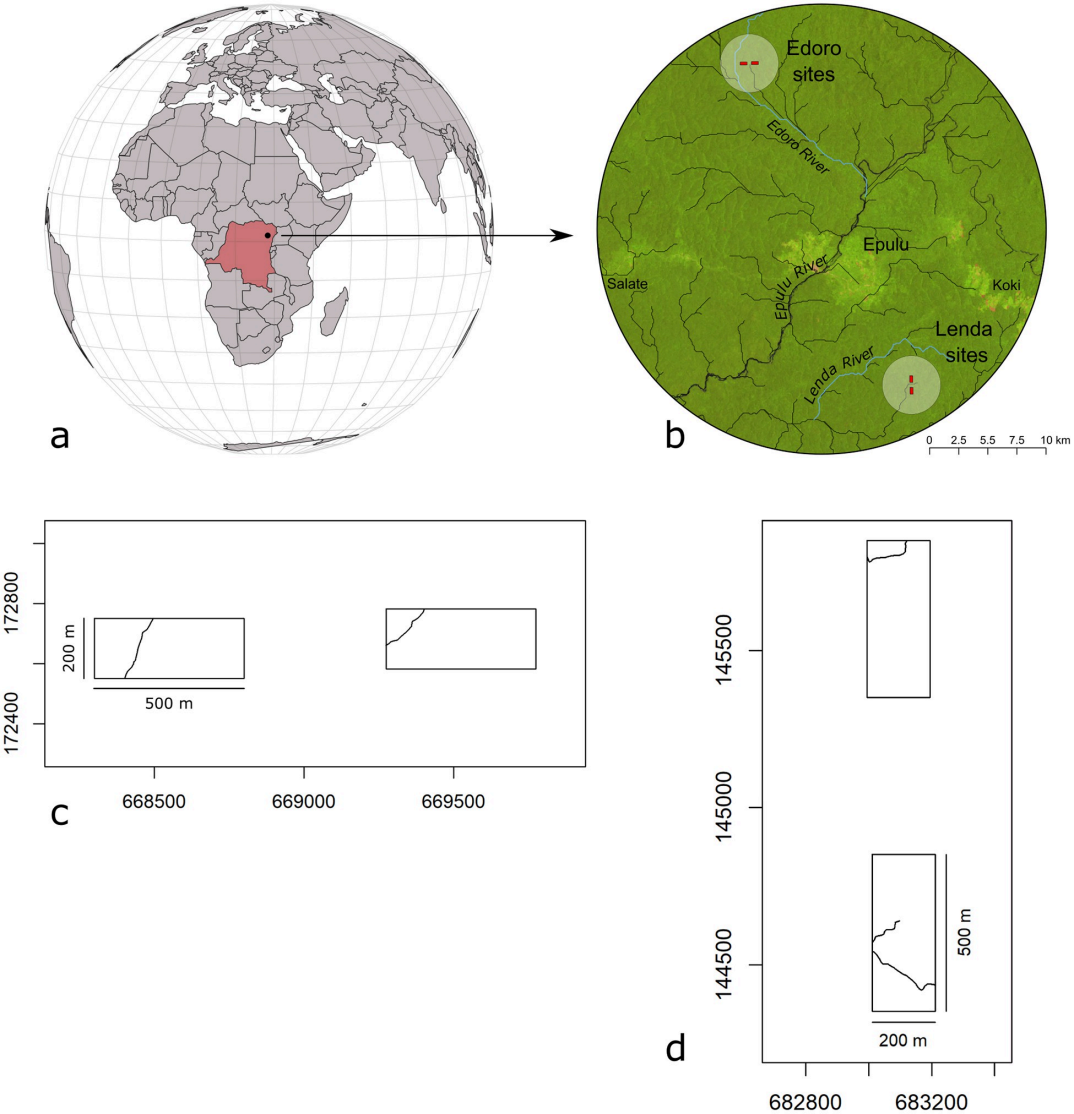
Overview of study area and Ituri Forest Dynamics Project field plots. The location of Ituri field plots in the DRC (a); regional context, showing the two sets of field plots, named for their proximity to the Edoro and Lenda Rivers (b); and local context, showing Edoro (c) and Lenda (d) field plots with minor plot-level hydrological features digitized from [29]. The eastern Edoro plot was omitted from analysis due to lack of *Gilbertiodendron*. Basemap from Global Land Analysis & Discovery laboratory 2019 Landsat mosaic. Regional hydrological features derived through hydrological analysis of 3 arc second Shuttle Radar Topography Mission digital elevation tile North 01 East 28.

This research was based on three of four field plots associated with the Ituri Forest Dynamics Project, which have described in detail by [13, 29] and references therein. Established in 1994 by the Centre de Formation et de Recherche en Conservation Forestière and jointly managed with the Smithsonian Institution’s Center for Tropical Forest Science (CTFS), the Wildlife Conservation Society (WCS), and the Okapi Faunal Reserve (OFR), the Project includes four 10-ha (200 m × 500 m) field plots covering the range of local forest types: mixed species forest, *Gilbertiodendron*-dominated forest, transitional forest between mixed and monodominant, and forest associated with riparian zones or other microsites. The field plots occur in two groups named for the Edoro and Lenda Rivers: edodo1, edoro2, lenda1, and lenda2. The edoro2 field site was originally created as a control plot. At the time of installation, it was composed entirely of mixed species forest and did not contain any *Gilbertiodendron*. For this reason, it was omitted from the present analysis. The remaining field plots fall on acidic, well-drained oxisols at 700–850 m above sea level, with limited topographic variability (mean of 18 m between lowest and highest points) [4, 29] (Fig 1).

Of particular interest here, the Ituri Forest Dynamics data includes repeated measurements for all stems ≥ 1 cm DBH (diameter at 1.3 m height). From Feb. 2, 1994—Jul. 15 1996, Feb. 2, 2001—Jan. 9, 2002, and Jan. 10—Nov. 30, 2007, three separate censuses were carried out in which every stem was tagged, identified (> 99% to the species level), measured for DBH, and geographically referenced to a local origin. A fourth census was conducted between Aug. 1, 2015 and Apr. 17, 2018, but at the time of this analysis, the data had not been sufficiently cleaned and unified to permit its inclusion in the present work. Our full tabular dataset included 959,312 individual stem measurements from 342,893 unique trees across the four field sites and three censuses. Of these, we relied on 34,499 records of living *Gilbertiodendron* with valid DBH or BA measurements, and omitted 1,411 records of dead stems lacking suitable diameter measurements. All field plots with *Gilbertiodendron* contained small diameter seedlings and saplings (≤ 2 cm DBH) at each census, though the number of small stems was an order of magnitude greater on the lenda field plots (*n* = 1151–1910 per census), than on the edoro1 plot (*n* = 158–182 per census). Where *Gilbertiodendron* stands display synchronous supra-annual masting every 3–5 years [4, 18], the small stems might appear to implicate local masting during inter-census years. However, *Gilbertiodendron* growth is quite slow in shaded understory conditions, and small stems are generally older than they appear [18].

For those of our analysis that required locational information, it was necessary to record geospatial (*x*,*y*) locations for each stem. For the 782 records (2%) that lacked coordinate information due to issues with record keeping, we were able to identify each stem’s 20 m × 20 m parent quadrat (a function of the field study design), and then randomly situated each stem within that quadrat. Randomization was performed across both *x* and *y* dimensions, and ensured that no two stems were located at the same location. This technique provided unbiased coordinates in aggregate, and suited our needs given the spatial distribution of coordinate-deficient records and the suite of techniques used in subsequent analyses. To move stem records from a local geospatial referencing system to global coordinate system, spatial field plot boundaries were defined as 200 x × 500 m rectangles based on plot origins, which were themselves established using a civilian-grade GPS transceiver. We initially defined the plot corners with respect to the World Geodetic System of 1984 (WGS84), and then projected them to Universal Transverse Mercator Zone 35 North (UTM 35N, m). To covert each stem’s original coordinates into the projected coordinate system, we then applied stem-specific offsets relative to each plot origin.

### Monodominant patch delineation

**Fig 2:**
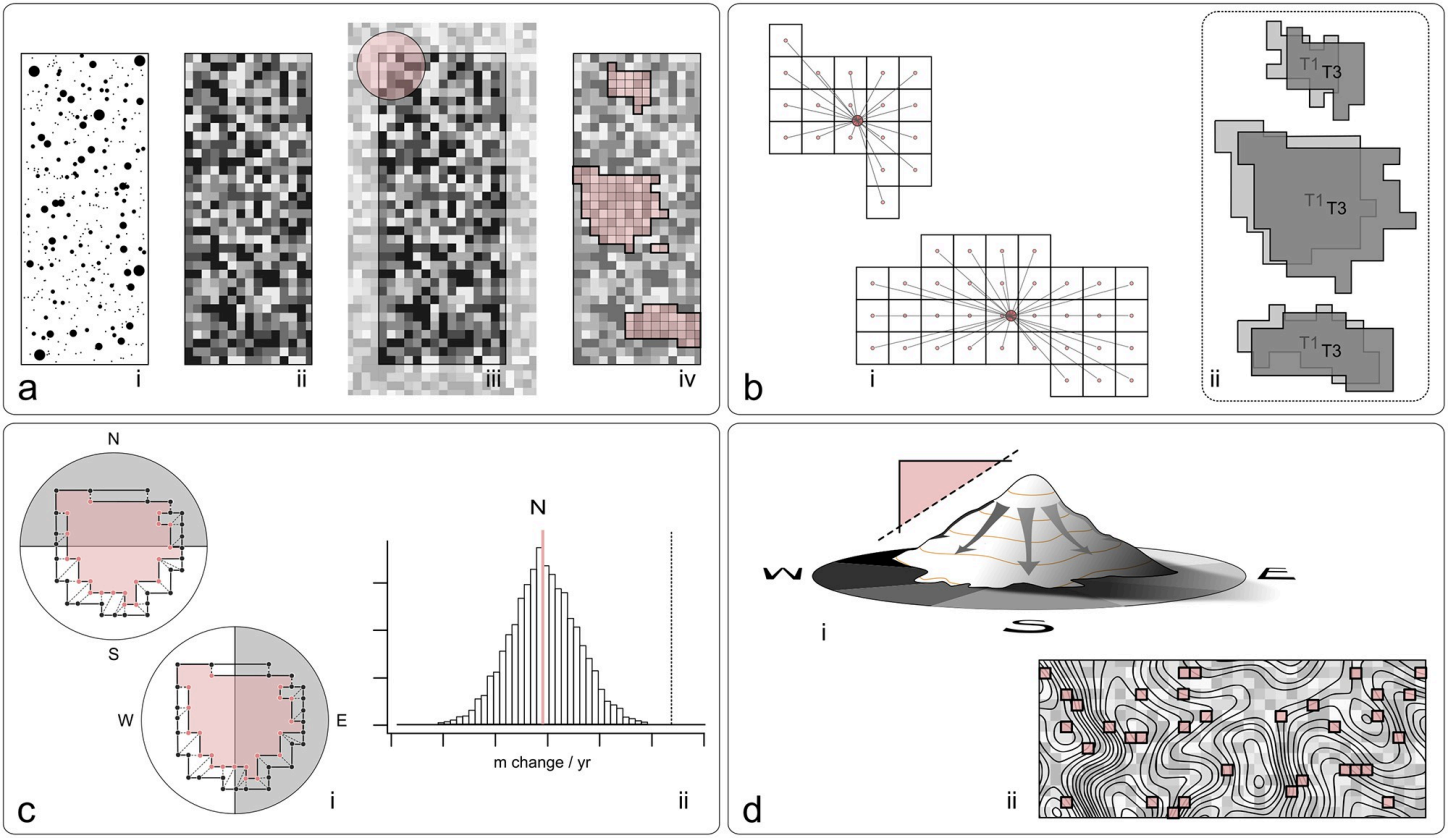
Conceptual diagram of methods employed in this study. (a) Raw tabular data was cleaned and coerced into spatial format (i). Basal area was aggregated to grid cells (ii), whose values were minimally smoothed after accounting for edge effects (iii). Monodominant forest patch delineation was based on isolines of BA intensity (iv). Three different different analytical approaches were then used to evaluate spatial propagation of monodominant forest on each field plot (b-d). (b) Scale-independent radius of gyration was computed for each patch (i), and a patch area-weighted mean was computed for each field plot (ii). The final metrics were used to evaluate change between the first and third censuses. (c) Patch vertices from the first and third censuses were paired based on measures of proximity (i). Regression was used to look at changes in latitude and longitude for each of four cardinal quadrants (i). Permutation testing was used to determine if the linking algorithm was producing demonstrable, non-random measures of change (ii). (d) The difference in basal area intensity between the first and third censuses was treated as a topographic surface from which vector magnitude (slope) and direction (aspect) were derived (i). Spatial bootstrapping of the pixelated difference surfaces was used to evaluate plot-level gains or losses across time (ii).

To model the translation of *Gilbertiodendron*-dominated forest over space, we employed three distinct strategies. In the first, we considered changes in the area-weighted mean radius of gyration over both space and time. In the second, we used linear models to consider changes in the projected coordinates of patch boundaries over space and time. In the third, we used vector fields to visualize and quantify the magnitudes and directions of change in *Gilbertiodendron* basal area (BA) intensity over space and time (Fig 2).

To understand how monodominant forests were changing, we first isolated records from each field site and census that contained *Gilbertiodendron dewevrei* (*n* = 9, as edoro2 had no *Gilbertiodendron* by design). For each of these subsets, we used the spatstat package [30, 31] to develop a continuous surface of aggregated *Gilbertiodendron* BA within 1 m^2^ grid cells. Prior to applying a moving-window function to each grid, we implemented an edge correction strategy drawn from the sampling literature. When measuring the contents of a field plot that borders the parent domain, there are implicit edge effects from the portions of plots that extend beyond the measurable domain. Gregoire and Valentine [32] discuss the “reflection method” as one method to account for these effects. In the reflection method, a direction vector from the plot origin to the domain boundary is established, and then “the direction vector is folded back over itself at the boundary and the ‘reflected plot’ is installed where the vector terminates inside of” the initial plot. Here, we employed the reflection method, but instead of reflecting the plot—equivalent here, to each of the moving windows described below—to make up for the portion that falls outside domain boundary, we reflected the BA intensity surface. Reflection was performed in each of the cardinal and inter-cardinal directions to produce, for each field site, a continuous grid 200 m larger than the original on all sides.

To the expanded BA surface, we used the raster package [33] to apply a circular moving-window summation with radius 56.42 m, capturing the per hectare BA within the focal neighborhood [34] of each cell. To these grids, a 3 × 3 cell moving window mean was applied to reduce the sinuousity and vertex complexity of the margins of forest patches (described below). These grids, one for each of three sites and two time periods (census 1 and census 3 only), were then cropped to their original dimensions. From these surfaces of BA per hectare, we identified *Gilbertiodendron*-laden forest patches for use in subsequent analyses. From the BA intensity surfaces, we generated isolines corresponding to 20 m^2^ of BA on the lenda1 and lenda2 field sites, and the isolines corresponding to 10 m^2^ of BA on the edoro1 field site (since it had substantially less well-developed *Gilbertiodendron* dominance compared to lenda1 and lenda2). While the Ituri rainforest exemplifies monodominant and mixed species forest assemblages, at times with clear boundaries [16, 22], the interspersion and juxtaposition of *Gilbertiodendron*-dominated forest against other forest types is not so clearly defined within our field data. We selected these 20 m^2^ and 10 m^2^ thresholds based on an approximate, but ultimately subjective, assessment of what constituted patch boundaries. Finally, using functions from the raster package [33], isolines were converted into discrete (singlepart) polygonal features, adopting the boundary of the field site in cases where the two intersected. These features represented the *Gilbertiodendron*-dominated forest patches of interest.

### Measuring area-weighted mean radius of gyration

Our first approach for quantifying the movement of *Gilbertiodendron*-dominated forest patches was based on the area-weighted mean radius of gyration [35, 36] (1), which we computed for each field site and census. Also known as correlation length, this metric is often used to characterize animal movement. Keitt *et al.* [35] described it as the mean distance an individual (animal or otherwise) is able to move before reaching a barrier, when placed randomly within suitable habitat. Taking an area-weighted average of radius of gyration values essentially captures the average distance an individual might travel while remaining within the same patch [36]. While trees cannot move, we can treat offspring from a given tree as future versions of the seed-producing parent. This approach provided a speculative way to answer the question, if trees could move, where would we expect tree *x* to be located *n* years from now?

Area-weighted mean radius of gyration is directly related to landscape fragmentation, for as values increase, so too does connectivity of suitable habitat. In our usage, “barrier” is synonymous with the edge of a target patch, and by taking an area-weighted mean across each field site, we establish an intuitive measure of the site-wise spatial extent of patches. There are a number of metrics that seek to capture the spatial extent of land cover patches [36], and we elected to use area-weighted mean radius of gyration to avoid scale dependence. For each field site, we subtracted the area-weighted mean values associated with the first census from those of third census in order to understand, for each field site, the average annual landscape-level expansion or recession distance (in meters) of *Gilbertiodendron*-dominated patches. Radius of gyration is computed as

$$\begin{array}{c} \begin{array}{c} \text{radius}\;\text{of}\;\text{gyration}=\sum_{r=1}^{z}\frac{h_{ijr}}{z} \end{array} \end{array}$$ (1)

where *h*_*ijr*_ is the distance between cell *r* within patch *ij* and the centroid of patch *ij*, using cell-centroid-to-cell-centroid distance, and where *z* is the number of cells in patch *ij*. In practice, for each of our six collections of forest patches (three sites × two censuses), we computed the radius of gyration for each conterminous patch before using the patch areas to obtain an area-weighted mean. Patches as small as 1 m^2^ in size and containing as few as a single stem or coppice were considered, and an 8-cell (“queen”) adjacency rule was employed.

### Modeling changes in patch boundaries

In our second strategy for characterizing the movement of *Gilbertiodendron*-dominated forest patches, we used an algorithm to pair and trace patch boundary vertices over time and space, emulating organism mobility. We used the raster [33] and rgeos [37] packages to isolate only those patches ≥ 300 m^2^, and those whose nearest boundary segments were ≥ 30 m from another patch. Since we were interested in *established* forest patches, the former rule sought to mitigate the impacts of spurious individuals and small patches of *Gilbertiodendron* that may materialize and dematerialize over time through natural successional dynamics or disturbance processes. The latter rule was imposed in order to identify *unique* (i.e., spatially isolated) patches. Species in the *Gilbertiodendron* genus tend to have deep crowns with poor seed dispersal [23]. The heavy seeds are ballistically dispersed upon dehiscence of the seed pods, limiting advancement across the landscape [18, 38]. Hart [18] identified seed dispersal distance to be 5–10 m beyond the vertical projection of the crown edge, and van der Burgt *et al.* [39] (see also [40]) speculated that the maximum distance “is probably in the range of 50–70 m” (p. 56). Van der Burgt [38] (p. 29) also speculated that the upper bound on dispersal distance from the tallest tree in his study was “likely around 60 m”. In light of our limited data, we elected to apply a conservative threshold of 30 m, beyond which we considered target features to be part of a unique patch. Patches that met these criteria were assigned unique identifiers in order to track them over time.

For each unique patch, we employed a matching algorithm to measure the distance between patch boundaries at the first and third censuses. Working with patch boundary vertices (as opposed to inter-vertex line segments, or edges), we matched each vertex from the third census to the nearest vertex from the same patch in the first census in order to link vertices based on the most likely movement trajectory for each point on the patch boundary. Here, “most likely” was equated with the shortest Euclidean distance, which was, in most cases, approximately orthogonal to the boundary curve.

For each unique patch, a first pass of our vertex-matching algorithm moved sequentially through each vertex in the census 3 patch boundary, identifying the closest vertices within the census 1 boundary. The census 3 vertices were then ordered by distance to their matching vertices, and the pairing algorithm was applied to all census 3 vertices in descending order of distance. Because *Gilbertiodendron* patches generally expanded over time, there were more vertices in the census 3 patch boundaries than in their census 1 counterparts. For this reason, we permitted up to two vertices in the third census to be paired with the same vertex in the first census on the lenda1 and lenda2 field sites (≤ 2 : 1), and, because of even greater expansion, up to three vertices from third census on the edoro1 field site (≤ 3 : 1).

For each unique patch in each field site, paired vertices were grouped according to their cardinal quadrants relative to the census 1 patch centroid. Pairings were assigned to east or west quadrants based on a north-south longitudinal meridian cutting through the patch centroid, and to north or south quadrants based on an east-west parallel cutting through the patch centroid. Defining locational quadrants in this way enabled us to use weighted simple linear regression (nlme::gls [41]) to model the change in projected coordinates of the paired vertices as a function of time, with one model for each quadrant, for each target patch, and for each landscape (*n* = 4 directions × 5 unique patches in total = 20 separate regressions). The regression models we employed can be expressed as,

$$\begin{array}{c} \begin{array}{cc} Y_{ijq} & =\beta_{0}+\beta_{1}year_{ijq}+\epsilon_{ijq}\\ Var(\epsilon_{ijq}|year_{ijq}) & =\sigma^{2}\delta_{s_{ijq}}^{2} \end{array} \end{array}$$ (2)

where *Y* = latitude or longitude, *i* = 1, …, *n* indexes (pairs of) vertices associated with the third census, *j* = 1, …, 5 indexes unique patches, and *q* = N, E, S, W captures the directional quadrant. Here, we employ a sub-model for the variance using nlme::varIdent [41, 42]. In the generic form, this enables the model to estimate a different *σ*^2^ for each level of a stratification variable *s*, indexed with *s* = 1, …, *S*. To paraphrase Pinheiro and Bates [41] (p. 209), this variance models uses *S* + 1 parameters to represent the *S* variances, requiring that we impose restrictions on the *δ* parameter to ensure identifiability. In all cases, *δ* > 0, *δ*_1_ = 1, and each subsequent *δ* value (2, …, *S*) represents the ratio of the standard deviations of the *s*th stratum and the first stratum. In the present application, we have only two strata; *s* = 1 for census 1 and *s* = 2 for census 3. After applying (2) to each quadrant of each unique patch, we extracted the model coefficients in order to identify the linear distance of change (meters) in the *Gilbertiodendron* patch boundary per year.

We employed case resampling-based bootstrapping (*n* = 10, 000) to compute estimates of mean annual change and the associated bias-corrected, accelerated 95% confidence intervals [43] (boot::boot.ci). Since our pairing algorithm was based more on ecological principles and common logic than common analytical strategies, we also used permutation testing to ascertain whether the results obtained were the product of a non-random and potentially meaningful process. The version of the pairing algorithm we implemented in our permutation tests was identical to that described above, except that we held the vertices from the third census constant and randomly permuted those of the first census. Thus, matched pairs were not necessarily the geographically closest. We computed 10,000 random permutations for each patch within each field site using cluster-based parallel computing. The results were aggregated and the distributions of coefficients for the *year* predictor were used to evaluate the statistical significance of our initial findings.

#### Quantifying the flow of basal area

Our third approach for characterizing the movement of *Gilbertiodendron*-dominated forest patches utilized vector fields. We used spatstat [30, 31] to convert our original stem locations to ppp objects (spatially bounded two-dimensional point patterns) with BA as marks, and then subjected these ppp objects to kernel intensity estimation using density.ppp.

We employed a multivariate Gaussian kernel, the Jones boundary correction [44], and a 25 m bandwidth. While we created BA intensity surfaces for our radius of gyration calculations, these relied on the empirical BA ha^−1^ around each pixel and were therefore insufficiently smooth for vector field analysis. Using the kernel smoothed intensity surfaces, we subtracted that of the first census from that of the third to obtain a difference grid. The difference grid is a three dimensional topographic surface to which we applied slope and aspect calculations for each 1 m^2^ cell on the landscape. The slope and aspect dimensions represent vector magnitude and direction, respectively. Using custom R functions, we plotted vector fields of change in BA intensity for each of the three field sites. To enhance interpretative clarity we uniformly scaled the vector magnitudes and visualized a systematic sample of vectors to avoid graphic overcrowding.

In addition to the vector field plots, we utilized a bootstrapping procedure to estimate the mean change in BA ha^−1^. To each of the three difference grids, we applied the same reflection-based edge correction method discussed above [32]. To the expanded grids we applied a circular moving-window summation (radius 56.42 m) in order to capture the empirical sum of the change in BA in 1 ha neighborhoods surrounding each pixel. Each of these grids was then reduced to its original size before using pixel-wise case resampling-based boostrapping (n = 10,000) to obtain a mean and 95% confidence interval for change in BA ha^−1^ within each field site.

## Results

When calculating differences in the area-weighted mean radius of gyration for different field sites containing *Gilbertiodendron*, we found that all patches were expanding (Table 1). The edoro1 field site was characterized by younger, less well established patches of *Gilbertiodendron* that occupied between 11.1% (census 1) and 15.1% (census 3) of the landscape. Over the 12-year study, these patches expanded at a rate of 0.44 m per year. Lenda1 had a single well-established patch that covered between 65.6% and 68.0% of the site, a 3.5% change equating to a patch expansion rate of 0.27 m per year. Lenda2 presented two well-defined patches occupying the majority of the site (≈82%). Advancement over time was positive but negligible, with a growth rate of 0.03 m per year.

**Table 1:** Area-weighted mean radius of gyration measures for *Gilbertiodendron*-dominated forest patches.

Site	Census 1 (%)	Census 3 (%)	% Increase	$\vec{\Delta }$ m yr^−1^
edoro1	11.4	15.1	33.2	0.44
lenda1	65.6	68.0	3.52	0.27
lenda2	82.1	82.4	0.35	0.034
Mean			12.36 (14.8)	0.25 (0.17)
Median			3.52	0.27

$\vec{\Delta }$ m yr^−1^ = expansion rate in meters per year between census 1 (1994–1996) and census 3 (2007), as computed from radius of gyration (1). Parenthetical values are standard deviations.

**Fig 3:**
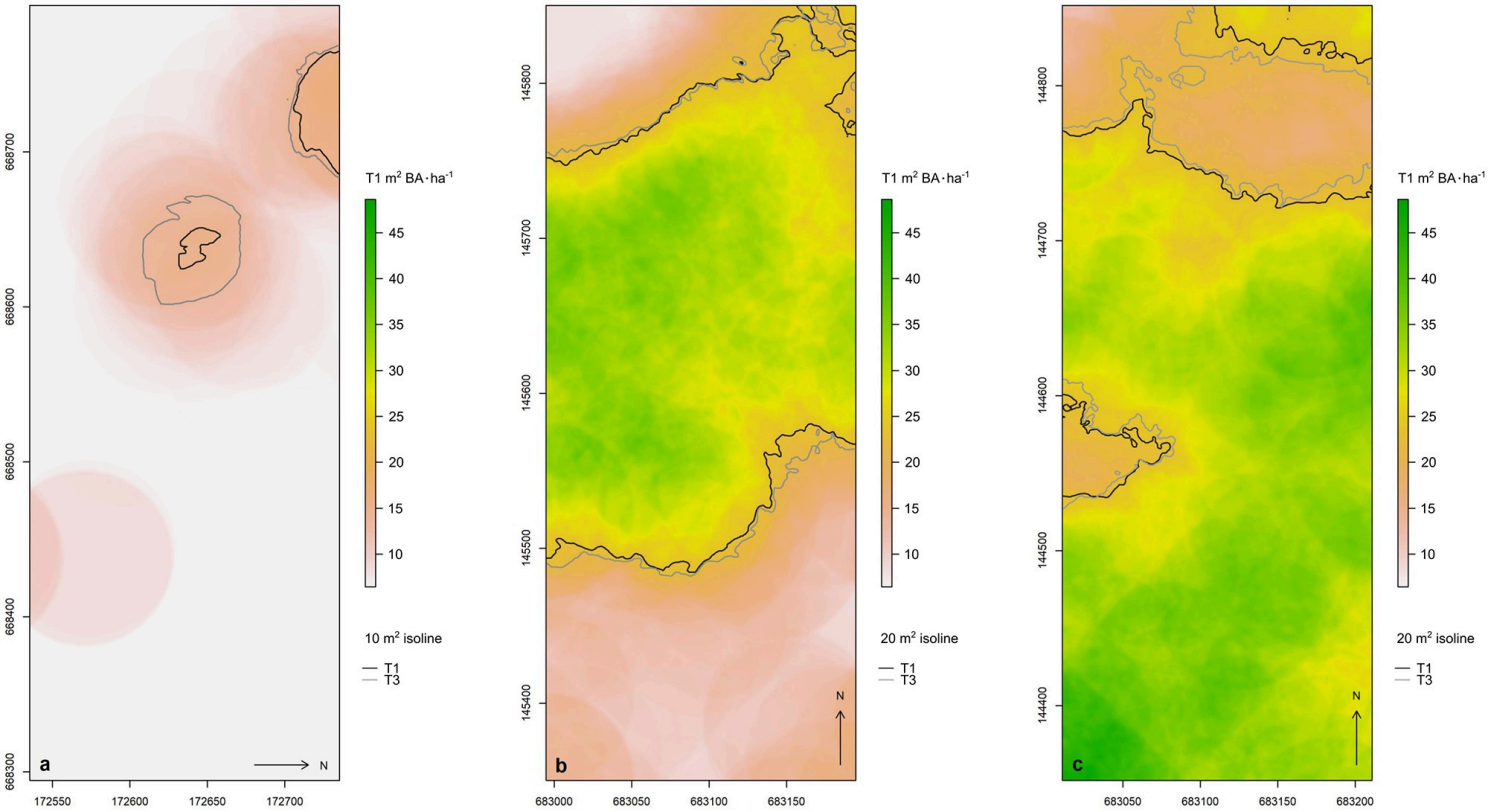
The movement of isolines of basal area intensity (m^2^ BA ha^−1^) across time. (a) edoro1 with 10 m^2^ isoline, (b) lenda1 with 20 m^2^ isoline, (c) lenda2 with 20 m^2^ isoline. T1 = 1994/1995, T3 = 2007. All coordinates are in UTM 35N (m).

Our isoline movement models generated comparable, if more nuanced, results. Fig 3 visually illustrates changes in *Gilbertiodendron*-dominated forest patch boundaries over the 12-year study period. Results were most pronounced in edodo1, whose patches were less mature than those of lenda1 or lenda2. The lenda2 field site showed minor recession towards the western margin of the site, but clear expansion into the mixed species forest median that formerly separated two distinct *Gilbertiodendron* patches in the north. The lenda1 field site showed minor expansion in the northeast, southeast, and southwest, but otherwise exhibited a more steady state than the other two sites. Along the largely coincident patch margins of lenda1 was a natural fluctuation with both expansion and contraction on the order of meters to tens of meters.

Table 2 presents the numerical results of our isoline movement modeling. Here, our expectation was that all patches would be expanding at statistically significant rates. Because we were modeling coordinates, and because all of the examined patches fell to the north and east of the UTM 35N coordinate system origin, patch expansion was characterized by increasing latitude in the northern quadrant (positive estimated coefficients, or + in the Expect. column), increasing longitude in the eastern quadrant (+), decreasing latitude in the southern quadrant (-), and decreasing longitude in the western quadrant (-). In aggregate, we found that 88% (7 of 8) of quadrants in edoro1 were expanding in accordance with expectations. This number is reduced to 75% (3 of 4) for lenda1 and to 63% (5 of 8) for lenda2. Thus, 75% (15 of 20) of quadrants reflect aggregate expansion of *Gilbertiodendron* into areas of (previously) lesser *Gilbertiodendron* BA intensity. The lenda1 field site did not exhibit any statistically significant changes, as can be seen in Fig 3. Meanwhile, 75% (6 of 8) of quadrants in edoro1 and 50% (4 of 8) of quadrants in lenda2 presented statistically significant patch boundary movement. Of the changes that were significant, only two (edoro1 patch 1 N, lenda2 patch 1 E) reflected recession instead of expansion.

**Table 2:** Bootstrapped results (*n* = 10, 000) of *Gilbertiodendron*-dominated forest patch boundary movement modeling.

Site	Patch	Quad.	L	M	H	< *α*	Expect.	Expan.
edoro1	1	N	-0.29	-0.15	-0.01	y	+	n
edoro1	1	S	-0.38	-0.24	-0.10	y	-	y
edoro1	1	W	-0.56	-0.17	0.21	n	-	y
edoro1	1	E	-0.13	0.17	0.46	n	+	y
edoro1	2	N	0.69	0.90	1.08	y	+	y
edoro1	2	S	-1.17	-0.94	-0.69	y	-	y
edoro1	2	W	-1.07	-0.87	-0.64	y	-	y
edoro1	2	E	1.25	1.42	1.59	y	+	y
lenda1	1	N	-0.29	0.05	0.37	n	+	y
lenda1	1	S	-0.78	-0.29	0.21	n	-	y
lenda1	1	W	-0.53	0.00	0.55	n	-	n
lenda1	1	E	-0.25	0.04	0.31	n	+	y
lenda2	1	N	0.43	1.27	2.12	y	+	y
lenda2	1	S	-1.48	-0.02	1.44	n	-	y
lenda2	1	W	-0.01	0.24	0.49	n	-	n
lenda2	1	E	-1.93	-1.21	-0.52	y	+	n
lenda2	2	N	-0.64	-0.32	0.01	n	+	n
lenda2	2	S	-0.70	-0.52	-0.31	y	-	y
lenda2	2	W	-1.59	-1.16	-0.77	y	-	y
lenda2	2	E	-0.57	0.01	0.59	n	+	y

L, M, and H are the estimates of the lower 95% confidence interval boundary, mean, and upper 95% confidence interval boundary estimates of site-, patch-, and quadrant-specific rates of movement (m/y). < *α* = are the model results significant at the 95% confidence level? Expect. = expectation of estimated coefficient sign, where hypothesized patch expansion is captured by positive coefficients (+) in N and E quandrants, and negative coefficients (-) in S and W quandrants. Expan. = y, yes patch expansion was observed, or n, no patch expansion was observed.

Since the prevailing wind patterns in the Ituri province are from the northeast, we might hypothesize that we would see greater expansion in the southerly and westerly directions than we would in northerly or easterly directions if the trajectories of ballistically dispersed seeds were being influenced by the wind. At both edoro1 patch 1 and lenda2 patch 2, the greatest rates of expansion were in the south and west quadrants. In edoro1 patch 2, the second and third greatest rates were in the south and west quadrants, and in lenda2 patch 1, the second greatest rate was in the south quadrant (the west had negligible change). The sole patch at lenda1 showed mixed results. When averaging across like quadrants from different field sites, the greatest rates of expansion were to the south and west (Table 2).

With few exceptions, our permutation tests (S1–S3 Figs) demonstrate that the algorithm we used to associate *Gilbertiodendron*-dominated forest patch boundaries in the first census to those in the third, identified non-random and potentially meaningful associations. The only test for which our original modeled results were not highly significant (*p* ≤ 0.0008) was that for the western quadrant of lenda2 patch 2. The probability of obtaining this particular model’s estimated coefficient was 0.06. This patch was the smaller of the two lenda2 patches, and the limited number of vertices present in the isoline boundary may have led to this marginally significant result.

**Fig 4:**
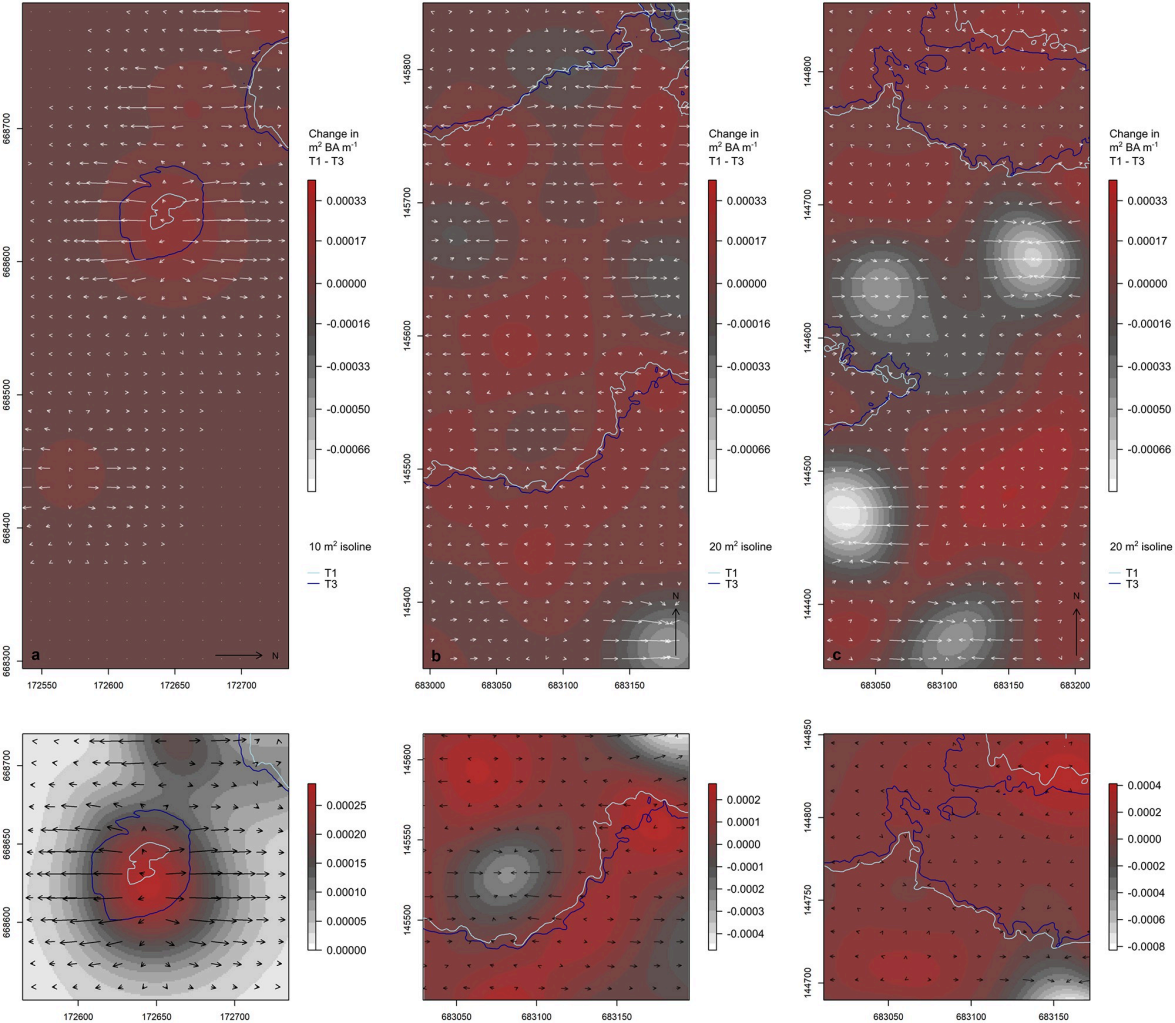
Net change in m^2^ of BA m^−1^ between 1994/1995 and 2007. Overview (upper) and details (lower; select examples) of vector fields illustrating change in m^2^ of BA m^−1^ between the first (1994/1995; T1) and third (2007; T3) censuses, for edoro1 (a), lenda1 (b), and lenda2 (c). Arrow orientation conveys the direction of change (aspect of the surface), while the length of each arrow conveys the magnitude of change (slope of the surface). A systematic sample of vectors is shown for graphical clarity, and vector magnitudes have been uniformily scaled to improve visual clarity. Note the different color ramp value ranges in the lower panels. All coordinates are in UTM 35N (m).

Fig 4 presents the results of our vector field analysis, illustrating changes in the BA for each 1 m^2^ pixel between the first and third censuses. As expected, the vast majority of changes are positive, reflecting a net increase in BA over time. This is empirically captured in our bootstrap results: the edoro1 field site increased by an average of 0.35 ± 0.002 m^2^ BA ha^−1^, the lenda1 field site increased by an average of 0.84 ± 0.004 m^2^ BA ha^−1^, and the lenda2 field site increased by an average of 0.41 ±0.01 m^2^ BA ha^−1^. Areas of change contain larger magnitudes along the peripheries of high and low concentrations regions. This is reflected in shorter length vectors over the peaks and valleys in Fig 4, with longer length vectors on the slopes leading to and away from the valleys and peaks. We would expect the greatest rates of change to occur just beyond the isoline derived from the third census’ data, as these are the areas in most rapid transition. We would also expect vector directions to reflect an intuitive expansion away from the heart of each patch. The edoro1 field site provides the clearest example of this behavior (Fig 4a lower panel). The southern margin of the sole patch at lenda1 contains a band of increasing BA that parallels the expanding isolines (Fig 4b lower panel), and the northern-most patch at lenda2 also reflects positive gains and expansion along the patch margin (Fig 4c lower panel).

The detailed plots in the lower portion of Fig 4 reflect moderate agreement between the expanding isolines emphasized in Fig 3 and the directions and magnitudes of change. Discrepancies between these two analytical approaches can be traced to the underlying data. Our isoline results are based on minimally smoothed surfaces of empirical BA/ha (i.e., a uniform kernel), while our vector field results are based on kernel smoothed BA intensity surfaces built with Gaussian kernels and a 25 m kernel bandwidth. It is well known that kernel smoothing is sensitive to changes in the bandwidth parameter [30, 45], and we caution the reader in drawing too close an association between our isoline and vector field results. Kernel intensity estimation is generally regarded as a non-parametric technique for exploratory data analysis, and we offer our vector field findings primarily as useful heuristics.

## Discussion

Our three sets of results are in concordance and collectively describe a successional process in which *Gilbertiodendron dewevrei* infiltrates and displaces adjacent closed-canopy mixed species forest, ultimately leading to the spatial propagation and increasing presence of monodominant forest. Of particular interest here is the rate at which *Gilbertiodendron* is expanding into mixed species forest. To date, there has been limited research on the successional rates of closed-canopy tropical forests with monodominant climax phases, making it challenging to place our findings into a larger context. As points of comparison, we first draw on a number of studies examining tropical monodominant-mixed species interactions that still exist (§ Monodominant-mixed species interactions), before considering successional modes and advancement rates derived from other ecotones (§ Successional modes and rates of forest advance). We then consider gap dynamics in patch interiors, as they relate to *Gilbertiodendron*’s spatial propagation (§ Invasibility and gap dynamics), and end by highlighting the inherent limitations in our findings (§ Limitations).

### Monodominant-mixed species interactions

The idea that monodominant forest species possess unique characteristics that enable them to invade adjacent mixed species forests, arose nearly a century ago, if not earlier [46]. The *Mora excelsa*-dominated forests of Trinidad, which continue to invade the nearby Crappo-Guatecare forest, were an early focus [2, 46–49]. *Mora excelsa*’s high canopy presence [2], relatively sharp monodominant-mixed species boundaries [2], and dense sapling growth within colonization fronts [46], share parallels with the forests at Ituri. Eggeling [50] also presents an early work, in which he describes the slow invasion of a mixed species forest by *Cynometra alexandri* in the Budongo rainforest of Uganda. As at Ituri, the author describes several forest types that represent a successional trajectory, placing the mixed species forest as seral to the *Cynometra*-dominated climax forest (c.f., [13]). Eggeling [50] also describes how *Cynometra* appears before the mixed species forest has peaked, increasing pressure on other species and slowly asserting itself through understory growth, not unlike what has been observed with *Gilbertiodendron* [17, 21]. From these early works, Beard [2] provides us with a rare map (c.f. Fig 6) of speculated *Mora excelsa* advancement trajectories into mixed species forest.

Hart *et al.* [16] write of the same Ituri rainforest examined in the present study, describing the association between *Gilbertiodendron*-dominated forest and mixed species forest in relation to other tropical monodominant forest complexes. In line with Eggleling’s [50] observations, Hart *et al.* [16] present a case for mixed species forest as seral to *Gilbertiodendron*-dominated forest, and they view monodominant forest boundaries as a reflection of the slow dispersal of the monodominant species following reduction in its historical range by large-scale disturbance. They did not find any reason to think *Gilbertiodendron*, once established, would not continue to assert its dominance over multiple generations (i.e., Connell and Lowman’s [8] type I monodominance). Makana *et al.* [21] present similar findings for the Ituri sites.

Henkel [7] describes the life history and growth traits of *Dicymbe corymbosa*, a *Caesalpiniaceae* monodominant in the mountains of western Guyana. He identified a number of structural traits that mirror those at Ituri and which suggest a similar, though perhaps slower, set of successional dynamics. These traits include reduced stem densities, reduced species diversity, and increased basal area in monodominant plots relative to mixed species plots, as well as the complete absence of *Dicymbe* in mixed species plots. Like *Gilbertiodendron*, the growth characteristics of *Dicymbe* accentuate a type I monodominant [8] with a strong ability to gain and retain dominance through mast fruiting, high recruitment rates, litter trapping, an ectomyccorhzial association, conspecific suppression through shade, and a coppicing growth form that leads to “cumulative reiteration” [7, 51]. In nearby French Guiana, Charles-Dominique *et al.* [52] present a study on the colonization front of an understory palm, *Astrocaryum sciophilum*, at its interface with undisturbed lowland rainforest. Simulation studies revealed a clear pattern of heterogeneous advance, and field observations were used to estimate an advancement rate of 2.3 m year^−1^ for *Astrocaryum* into mixed species forest. To our knowledge, this is the only published rate of advancement of a colonizing tree (or tree-like) species within closed-canopy tropical rainforest.

Marshall [46, 47], Gunther [48], Beard [2], Eggeling [50], Hart *et al.* [16], and Glick *et al.* [13] all present examples in which mixed species forest is seral to monodominant forest. In contrast, Peh *et al.* [23] test the notion that *Gilbertiodendron* monodominance is seral to mixed species forest, perhaps building from Hart *et al.*’s [16] discussion of theoretical patterns of succession among adjacent monodominant and mixed species forest communities. Although they identified seven mixed species forest species succeeding in *Gilbertiodendron*’s understory, this is a relatively small percentage in relation to the 438 unique species at Ituri [13]. While we agree with Peh *et al.*’s [23] assertion that *some* species can establish themselves in *Gilbertiodendron*-dominated forest, we speculate that this may be a function of the transitional forest structure that exists at the interface between mixed species forest and monodominant forest [13]. Further, if *Gilbertiodendron*-dominated forest was a successional community, we would generally expect the species to possess the characteristics of an early successional pioneer [16]. Yet, to paraphrase Hart *et al.* [16], we have little to no evidence of a more-specialized species that can or will succeed *Gilbertiodendron* in the absence of major disturbance.

Similarities between different monodominant forests have been examined previously [16], and the above-referenced studies indicate that monodominant-mixed species associations also share a suite of characteristics. As at Ituri, transitional forest structures, limited monodominant presence in mixed species forest, climax monodominance, colonization fronts, shade tolerance, and the ability to retain dominance over more than one generation, are characteristics present in comparable forest associations. Only Peh *et al.* [23] provide any evidence of successional processes that question *Gilbertiodendron*-dominated forest as a climax state. Since only one of the above-referenced studies provides us with an estimated rate of a species’ advance into mixed species forest (neither a dominant nor canopy species), we now turn to forest/non-forest ecotones for additional insight.

### Successional modes and rates of forest advance

Although forest/non-forest ecotones share markedly different successional trajectories than those of the closed-canopy forest at Ituri, they provide some of the very few estimates of forest encroachment rates that we could locate. Forest/non-forest ecotones follow two primary modes of forest succession: (a) linear edge diffusion [53], in which succession occurs as an advancement front oriented roughly perpendicular to the progression trajectory; and (b) a more stochastic process of clump formation surrounding a nucleus of opportunistically established early successional flora, followed in turn by the coalescence of clumps that gain adjacency through edge diffusion [54, 55].

References to clump coalescent succession first appeared in the early 1960s, if not earlier. Hopkins [56] conjectured that rapidly spreading forest overtaking savanna in western Nigeria was a function of isolated forest foci all spreading out into the surrounding landscape. By comparing two hand-drawn maps (with error acknowledged by the author), he estimated the exceptional annual expansion rate of 33 m year^−1^. Whittaker *et al.* [57] and Archer [54, 58] described succession via opportunistic clump formation as a two-phase process in which there was an open continuous phase characterized by a grassy matrix, and a discontinuous phase in which the matrix was punctuated by clumps of woody vegetation. They examined a mesic grassland/savanna/forest ecotone in southern Texas, USA. (c.f. [58] Fig 1) for which the mean absolute growth rate across clusters of all sizes ranged from 1.8 to 3.2 m year^−1^ radially [54]. In their carbon isotope-based analysis of a savanna/forest ecotone in east-central Cameroon, Guillet *et al.* [59] described clump succession as non-linear and faster than edge diffusion. They felt that clump coalescence was the dominant successional process in their study area, and that it was historically responsible for relatively rapid colonization of the savanna. Puyravaud *et al.* [60] observed that vegetative thickets served as nodal centers in a successional process leading from grassland to rainforest in the Western Ghats of southern India and, if left undisturbed, thickets would coalesce to form secondary rainforest. Favier *et al.* [53] studied forest/savanna ecotones in coastal Republic of Congo and found both modes of succession at play in the conversion from savanna to forest.

Relative to clump succession, succession by edge diffusion may be easier to recognize in the field, and has been discussed many times over the years. Hopkins [56] described the fluctuating boundary of forest and savanna in western Nigeria, noting a forest encroachment rate of 3–4 m year^−1^. Citing Miège’s [61] work on savanna/forest boundaries in Côte d’Ivoire, Hopkins [62] refers to an expansion rate of 2.5–3.5 m year^−1^ over a decade or more. Schwartz *et al.* [63] focused on the Mayombe region of coastal Republic of Congo, and presented a case for at least four successive phases in a linear expansion that converted savanna to sparse secondary forest. In a second study of the same area [64], analysis of vegetation composition, vegetation structure, and soil organic carbon isotope ratios revealed that enclosed savanna was being overtaken by forest at a rate of 0.14 to 0.75 m year^−1^, with a mean rate of 0.2 to 0.5 m year^−1^. Delègue *et al.* [65] also used carbon isotope analysis in their work on savanna/forest mosaic of coastal Gabon. They estimated the current rate of encroachment to be approximately 1 m year^−1^. Fabing [66] (as cited in [53]) used aerial imagery to estimate that coastal forest was advancing into savanna at a rate of 1 m year^−1^ in the Republic of Congo. Favier *et al.* [53] also studied littoral Republic of Congo and observed that, in moving from forest towards savanna, there were progressively smaller trees and less shade-tolerant species, indicative of a colonization front. They estimated the edge diffusion rate of advancement to be approximately 1 to 2 m year^−1^ based on a model of diametrical growth of *Aucoumea klaineana*. Youta Happi [67] reported on the diffusion of forest patches into savanna in Cameroon between 1951 and 1996 (c.f., Fig 48). He found forest to be advancing between 0.6 and 2 m year^−1^, with certain forest edges advancing less than 0.25 m year^−1^. The author acknowledged the limitations in accuracy, and cautioned the reader in holding too strict an interpretation of these rates. Cuni-Sanchez *et al.* [68] studied savanna/forest transition and boundary dynamics in Central Gabon, where monodominant Okoume forest was a seral phase following a colonizing forest front. They did not quantify linear rates of advancement of any forest type, but found succession to be slow; after 20 years, not a single field plot could be characterized as the subsequent stage in their putative successional scheme.

We can see from Table 3 that our findings from Ituri are in line with some of the published rates of forest succession in Central Africa. In comparing all of our quadrant models (*n* = 20) to the range of forest advancement rates from across Central Africa, however, we find that the rates of expansion at Ituri are significantly slower than other sites (-0.75 ± 0.51/-∞ m yr^−1^, p = 0.01). In particular, there is a much lower expansion rate associated with the eastern Ituri quadrants (0.09 m yr^−1^). This is largely a function of a single outlying value which, when removed, increases the estimated expansion rate and narrows the confidence interval (Table 3). Still, the rates of expansion are significantly slower than other Central African sites (-0.67 ± 0.5/-∞ m yr^−1^, p = 0.02). Findings from Thomas *et al.* [27], whose field data is encapsulated by the presented study, have been included in Table 3 for reference. While Thomas *et al,* [27] looked over a more limited time period and their analytical approach differed from any of those used here, they presented a comparable rate of advance.

**Table 3:** Edge diffusion-based expansion rates in forest succession among different ecotones.

Ecotone	Location	Exp. Rate (m y^−1^)	Ref.	Notes
grassland → forest	Southern Texas, USA	1.8–3.2	[54]	*a*, mean range
savanna → forest	Mayombe region, Rep. of Congo	0.14–0.28	[64]	*a*, *b*, Makaba site
savanna → forest	Mayombe region, Rep. of Congo	0.27–0.75	[64]	*a*, *b*, Kwilila site
savanna → forest	Coastal Gabon	1	[65]	*a*, *b*
savanna → forest	Coastal Rep. of Congo	1	[66]	*a*, *b*
savanna → forest	Coastal Rep. of Congo	1–2	[53]	*a*, *b*
savanna → forest	Central Cameroon	0.6	[67]	*a*, *b*, north of Akonolinga
savanna → forest	Central Cameroon	1.2	[67]	*a*, *b*, between Bertoua and Batouri
savanna → forest	Central Cameroon	2	[67]	*a*, *b*, confluence of Mbam and Kim
savanna → forest	Central Cameroon	0.25	[67]	*a*, *b*, lower bound
savanna → forest	Western Nigeria	3.4	[56]	*a*, *b*, typical boundary
savanna → forest	Western Nigeria	33	[56]	Atypical boundary, derived from maps
savanna → forest	Côte d’Ivoire	2.5–3.5	[61]	*a*, *b*, cited in [62]
mixed → mixed + loc. abu.	Central French Guiana	2.3	[52]	*a*
mixed → monodominant	Ituri region, Dem. Rep. of Congo	0.34	[27]	Encapsulated by present study
mixed → monodominant	Ituri region, Dem. Rep. of Congo	0.35	*	*a*, *b*, mean of northward
mixed → monodominant	Ituri region, Dem. Rep. of Congo	0.09 (0.41)	*	*a*, *b*, mean of eastward (omits outlier)
mixed → monodominant	Ituri region, Dem. Rep. of Congo	0.4	*	*a*, *b*, mean of southward
mixed → monodominant	Ituri region, Dem. Rep. of Congo	0.39	*	*a*, *b*, mean of westward
mixed → monodominant	Ituri region, Dem. Rep. of Congo	0.31 ± 0.3 (0.39 ± 0.26)	*	Mean of present study (omits outlier)
Mean	Africa, Fr. Guiana, Southern USA	1.22 ± 0.54		Using *a*
Mean omitting [54]	Africa	1.06 ± 0.56		Using *b*

* = the present study; *a* and *b* = values used in respective averages (see Notes column in relation to overall mean values at bottom of table). Where ranges are presented, an average of the minimum and maximum was used in computing global means. Global means include ± 95% margin of error.

Overall, these findings are not unexpected given that, with the exception of Charles-Dominique *et al.*’s [52] work, the figures in Table 3 were not derived from undisturbed closed-canopy lowland rainforest communities. Our findings agree with previous research in the Ituri region suggesting that the dispersal of *Gilbertiodendron* is likely to be slow, given this species’ slow growth rates and the fact that it has the heaviest seeds of any local species [12, 15]. The primary mode of forest succession taking place in our field sites is edge diffusion, as is particularly visible in edoro1 (Fig 3a). However, the northern portion of lenda2 (Fig 3c) implies a clump coalescent successional process may also be taking place. Here, we observe a central nucleus of *Gilbertiodendron* that is gaining in BA and, in conjunction with the edge diffusion of the primary patches, may eventually lead to a *Gilbertiodendron* isthmus cutting through the mixed species forest. This type of forest structure, in which islands of one forest type exist within another, was observed by Hart [16] many years ago. Hart [18] observed that the highest rates of *Gilbertiodendron* seed survival occur just beyond conspecific mast zones, perhaps as a function of increased survival benefits through reduced predator presence. This implies that transitional zones at the intersection of mixed and monodominant forest types are where we are likely to see the greatest expansion of monodominant forest, and that edge diffusion may be a more common mode of succession than clump formation or coalescence. This seems evident from Fig 3 and from the shifting forest classes presented in Glick *et al.* [13].

Gandhi *et al.* [55, 69] provide an alternative, theoretical lens through which to consider our observations of monodominant patch expansion. They observed a “critical nucleus size” as a spatial size threshold above which a patch of the competitively superior species will continue to grow, and below which it is not apt to persist. They found that the curvature of the interface between interacting species helps dictate the future fate of the species (c.f. [55] Fig 3). Patches tended to take on circular forms over time, and perturbations in the propagation front tended to decay, returning the patch to its more stable circular form. This aligns with their findings on cluster size, as once a cluster reaches the critical nucleus size, its mean perimeter curvature is reduced, placing the nucleating species in the competitively advantageous position within the interaction zone. The larger the patch, the shallower the mean curvature, and the greater the competitive advantage of the superior species. The dynamics described by Gandhi *et al.* [55] seem at play in our data, and where *Gilbertiodendron* has a number of advantageous life history traits [17], it appears that the critical nucleus size for the species is quite small (e.g., Fig 3a). The forest structures at Ituri provide a promising location for future field tests of Gandhi *et al.*’s [55, 69] findings.

### Invasibility and gap dynamics

**Fig 5:**
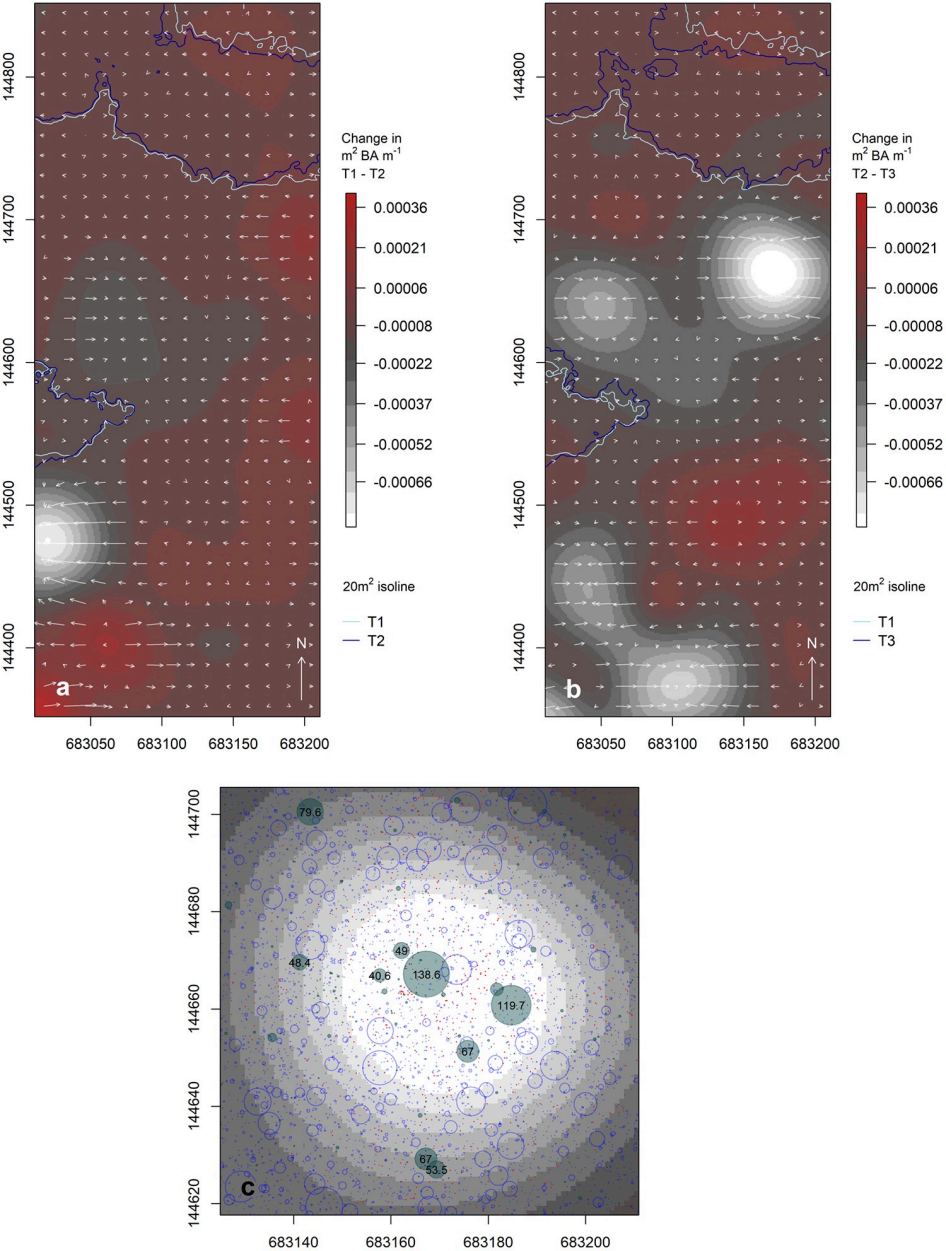
Gap dynamics on the lenda2 field site. Gap dynamics between (a) the first (1994–1995; T1) and second (2001–2002; T2) censuses, and (b) the second (2001–2002; T2) and third (2007; T3) censuses. See Fig 4 caption for vector field description. The most prominent gap visible on the eastern margin of (b) is detailed in (c), where blue circles represents stems present at the time of the first *and* second censuses, red dots represent stems recruited between the second and third censuses, and shaded circles represent stems lost between the second and third censuses. Stems ≥40 cm DBH have been labeled, and stem symbols have been scaled uniformly to ensure visual clarity. All coordinates are in UTM 35N (m).

The introduction, success, and range expansion of exotic species in a vegetative community is often associated with disturbance, whether natural or human-mediated [70]. This is true even of tropical rainforest communities where canopy gaps are the most common opportunistic channel for changes in species composition. Gap dynamics theory suggests that microclimatic conditions created by canopy openings are critical determinants of succession and invasibility within otherwise contiguous vegetative blocks [71, 72]. Yet, a number of studies [2, 23, 46–48, 50, 52], including the present one, suggest that exotic species can establish themselves in mature, closed-canopy tropical (and temperate [73]) forests in the absence of disturbance [23]. This is of interest in that we see both the propagation of *Gilbertiodendron* into closed canopy mixed species forest (Fig 4), and the formation of gaps in otherwise contiguous forested blocks (Fig 5).

The topographic depressions shown in Fig 4 reflect a general loss in BA across time, and highlight the way in which these landscapes are dynamic and vacillate in cumulative *Gilbertiodendron* presence. Using an example from the lenda2 field site, Fig 5 seeks to illustrate how gap dynamics actually manifest at Ituri. Here, we note the formation of one clear gap between the first and second census, as well as the formation of another set of gaps between the second and third censuses. Panel (c) of Fig 5 helps to illustrate the gap formation and closure process. The notable decline in BA between the second and third censuses, within the most pronounced gap, is primarily attributable to the loss of two stems (labeled), which were the two largest of those in the focal area. Pronounced changes in plot-level biometrics from select stem loss have been observed in other successional research [68]. In concert with stem loss, panel (c) shows high rates of recruitment (red points) in close proximity to these two lost stems.

There are a number of physiological and life history traits that enable *Gilbertiodendron* to advance into both closed and open canopy structures. These have been considered in greater detail elsewhere [9, 13, 15–18], but it is worth highlighting *Gilbertiodendron dewevrei*’s unique bimodal physiological response to light. Prolific reproduction and understory regeneration speaks to *Gilbertiodendron*’s ability to sustain itself under the deep shade of a closed canopy [18, 21], while the species has also shown an unexpectedly large growth response to strong light [19]. So, while the mature forests at Ituri demonstrate the gap dynamics we would expect of mature tropical forests, *Gilbertiodendron* is unusual in that it may be able to out-compete other late successional species during both the invasion of closed canopy forest and the recolonization of gaps [19].

### Limitations

One of the fundamental challenges with examining closed-canopy forest succession is that the forest margins of interest are not defined by clear boundaries such as those in a savanna/forest system. Thus, a choice has to be made in how the forest margin is located, and we recognize that our selection of isolines of 10 m^2^ and 20 m^2^ of BA introduces a level of abstraction. However, where BA intensity is a continuous spatial process, we argue that comparison of any BA intensity level that does not fall at the extremes of the field site (e.g., not in the lower or upper 10% of BA intensity values) will provide meaningful results. The relationship between tree age and BA accumulation is non-linear and quasi-asymptotic. Depending on what BA intensity is selected, the advancement rates might therefore appear different. However, changing the intensity of interest is equivalent to reframing the focus on the BA intensity associated with a particular stem age class.

Another challenge we face in quantifying the spatial propagation of shifting boundaries revolves around edge effects. Edge effects are well known and we have addressed them here using the reflection method [32] and the Jones boundary correction [44]. However, in our analysis of shifting isolines, we are still faced with the fundamental limits of the plot borders. From an analytical perspective, a *Gilbertiodendron*-dominated forest patch cannot expand beyond the plot border, effectively biasing our model-based estimates of advancement rates (e.g., Table 2, lenda2 patch 1 E). The solution to this problem would be to establish a long-term monitoring plot in which the spatial extent of the plot captured the entirety of a mixed species forest patch, such that estimates of encroachment of monodominant forest could be quantified using data contained entirely within the spatial domain of the study area. To some extent, our vector field plots (Fig 4) help to overcome this hurdle. They illustrate that, even at the margins of our field sites, BA intensity is increasing. This indirectly reflects the continued propagation of *Gilbertiodendron* outside of the measured spatial domain.

Finally, the present findings apply to long-term monitoring plots in a particular portion of the DRC. To better understand where our findings fall along a spectrum of common to exceptional *Gilbertiodendron* movement patterns, similar analyses would need to be conducted with data from other portions of *Gilbertiodendron*’s range. These efforts, drawing on local-level data, would be well supported by the spatially expansive view offered by remote sensing. Given the high cadence and historical span of available data products, analysis of moderate or high resolution multispectral imagery could offer rich insights into the propagation or contraction of larger monodominant patches. As additional high resolution data products enter the open market, we see this as a fruitful area of future research.

## Conclusions

The analyses presented here provide direct evidence of the slow advance of *Gilbertiodendron dewevrei* into mixed species forest in the Ituri region of Central Africa, and provide the first quantitative estimates of the rates of advance of a monodominant species within a closed-canopy tropical forest community. All of the analytical strategies we explored reflect that *Gilbertiodendron*-dominated forest patches are expanding in area and dominance (increasing BA). Our estimates of *Gilbertiodendron*’s encroachment rate on mixed species forest vary by cardinal direction, with a mean expansion rate of 0.31 m year^−1^ and greater rates of expansion in the direction of the prevailing winds. Mature patches of *Gilbertiodendron* present spatial structures typical of mature forest, with gap formation through loss of large stems and regeneration of new *Gilbertiodendron* recruits. The particular set of spatial analytical strategies we present provides a unique and complementary view of *Gilbertiodendron* dynamics that is not easily captured through alternative approaches, and we see continued utility in exploring tropical monodominance through a spatial lens.

## Supporting information

S1 Fig Permutation tests of isoline movement models for edoro1. Graphical results of permutation tests (*n* = 10, 000 iterations) of isoline movement models for site edoro1, patch 1 (a) and patch 2 (b). Histograms capture the null distribution of movement coefficients (m y^−1^) for each directional quadrant: N, S, W, E (clockwise from upper left). The black vertical line is the mean of the distribution, and the red vertical line captures the results of our observed algorithmic pairing of isoline vertices between the third and first censuses. All results highly significant at *α*=0.05.
(TIF)

S2 Fig Permutation tests of isoline movement models for lenda1. Graphical results of permutation tests (*n* = 10, 000 iterations) of isoline movement models for site lenda1, patch 1. Histograms capture the null distribution of movement coefficients (m y^−1^) for each directional quadrant: N, S, W, E (clockwise from upper left). The black vertical line is the mean of the distribution, and the red vertical line captures the results of our observed algorithmic pairing of isoline vertices between the third and first censuses. All results highly significant at *α*=0.05.
(TIF)

S3 Fig Permutation tests of isoline movement models for lenda2. Graphical results of permutation tests (*n* = 10, 000 iterations) of isoline movement models for site lenda2, patch 1 (a) and patch 2 (b). Histograms capture the null distribution of movement coefficients (m y^−1^) for each directional quadrant: N, S, W, E (clockwise from upper left). The black vertical line is the mean of the distribution, and the red vertical line captures the results of our observed algorithmic pairing of isoline vertices between the third and first censuses. All results highly significant at *α*=0.05, except patch 2 W (p = 0.06).
(TIF)

S1 Data R code for geospatial and statistical analysis. A compressed directory of R data science scripts, custom functions, and support files used to conduct the geospatial and statistical analyses presented herein.
(ZIP)

## References

[1] Davis T, Richards PW. The vegetation of Moraballi Creek, British Guiana: An ecological study of a limited area of tropical rain forest. Part II. The Journal of Ecology. 1934:106–155. doi: 10.2307/2256098

[2] Beard J. The Mora forests of Trinidad, British West Indies. The Journal of Ecology. 1946:173–192. doi: 10.2307/2256464

[3] Gérard P. Etude Ècologique de la Forêt Dense à *Gilbertiodendron dewevrei* dans la Région de l’Uele. Institut National pour l’Ètude Agronomique du Congo; 1960.

[4] Hart TB. The ecology of a single-species-dominant forest and of a mixed forest in Zaire, Africa. Dissertation, Michigan State University, Department of Botany and Plant Pathology; 1985.

[5] Makana JR, Terese B, Hibbs D, Condit R, Losos E, Leigh E. Stand structure and species diversity in the Ituri forest dynamics plots: A comparison of monodominant and mixed forest stands. In: Tropical Forest Diversity and Dynamism: Findings from a Large-Scale Plot Network. Chicago, USA: University of Chicago Press; 2004. pp. 159–174.

[6] Day M, Baldauf C, Rutishauser E, Sunderland TC. Relationships between tree species diversity and above-ground biomass in Central African rainforests: Implications for REDD. Environmental Conservation. 2013;41(1):64–72. doi: 10.1017/S0376892913000295

[7] Henkel TW. Monodominance in the ectomycorrhizal *Dicymbe corymbosa* (*Caesalpiniaceae*) from Guyana. Journal of Tropical Ecology. 2003;19(4):417–437. doi: 10.1017/S0266467403003468

[8] Connell JH, Lowman MD. Low-diversity tropical rain forests: Some possible mechanisms for their existence. The American Naturalist. 1989;134(1):88–119. doi: 10.1086/284967

[9] Hart TB. Monospecific dominance in tropical rain forests. Trends in Ecology & Evolution. 1990;5(1):6–11. doi: 10.1016/0169-5347(90)90005-X 21232309

[10] Read J, Hallam P, Cherrier JF. The anomaly of monodominant tropical rainforests: Some preliminary observations in the *Nothofagus*-dominated rainforests of New Caledonia. Journal of Tropical Ecology. 1995;11(3):359–389. doi: 10.1017/S026646740000883X

[11] Chave J, Condit R, Muller-Landau HC, Thomas SC, Ashton PS, Bunyavejchewin S, et al. Assessing evidence for a pervasive alteration in tropical tree communities. PLoS Biology. 2008;6(3):e45. doi: 10.1371/journal.pbio.0060045 18318600 PMC2270308

[12] Tovar C, Harris DJ, Breman E, Brncic T, Willis KJ. Tropical monodominant forest resilience to climate change in Central Africa: A *Gilbertiodendron dewevrei* forest pollen record over the past 2,700 years. Journal of Vegetation Science. 2019;30(3):575–586. doi: 10.1111/jvs.12746

[13] Glick HB, Umunay P, Makana JR, Thomas SC, Reuning-Scherer JD, Gregoire TG. Successional dynamics of *Gilbertiodendron dewevrei*Â drivesÂ forest structure and biomass in the Eastern Congo basin. Forests. 2021;12(6):738.

[14] Hart TB, Hart JA, Dechamps R, Fournier M, Ataholo M. Changes in forest composition over the last 4000 years in the Ituri basin, Zaire. In: van der Maesen LJG, van der Burgt XM, van Medenbach de Rooy JM, editors. The Biodiversity of African Plants. Wageningen, The Netherlands: Springer, Dordretch; 1996. p. 545–560. Available from: https://link.springer.com/chapter/10.1007/978-94-009-0285-5_70.

[15] Torti SD, Coley PD, Kursar TA. Causes and consequences of monodominance in tropical lowland forests. The American Naturalist. 2001;157(2):141–153. doi: 10.1086/318629 18707268

[16] Hart TB, Hart JA, Murphy PG. Monodominant and species-rich forests of the humid tropics: Causes for their co-occurrence. The American Naturalist. 1989;133(5):613–633. doi: 10.1086/284941

[17] Hall JS, Harris DJ, Saltonstall K, Medjibe VdP, Ashton MS, Turner BL. Resource acquisition strategies facilitate *Gilbertiodendron dewevrei* monodominance in African lowland forests. Journal of Ecology.2020;108(2):433–448. doi: 10.1111/1365-2745.13278

[18] Hart TB. Seed, seedling and sub-canopy survival in monodominant and mixed forests of the Ituri Forest, Africa. Journal of Tropical Ecology. 1995;11(3):443–459. doi: 10.1017/S0266467400008919

[19] Makana JR, Thomas SC. Effects of light gaps and litter removal on the seedling performance of Six African Timber Species. Biotropica: The Journal of Biology and Conservation.2005;37(2):227–237. doi: 10.1111/j.1744-7429.2005.00030.x

[20] Maley J. A catastrophic destruction of African forests about 2,500 years ago still exerts a major influence on present vegetation formations. IDS bulletin. 2002;33(1):13–30. doi: 10.1111/j.1759-5436.2002.tb00003.x

[21] Makana JR, Ewango CN, McMahon SM, Thomas SC, Hart TB, Condit R. Demography and biomass change in monodominant and mixed old-growth forest of the Congo. Journal of Tropical Ecology. 2011;27(5):447–461. doi: 10.1017/S0266467411000265

[22] Peh KSH, Sonké B, Lloyd J, Quesada CA, Lewis SL. Soil does not explain monodominance in a Central African tropical forest. PLoS One. 2011;6(2):e16996. doi: 10.1371/journal.pone.0016996 21347320 PMC3037391

[23] Peh KSH, Sonké B, Séné O, Djuikouo MNK, Nguembou CK, Taedoumg H, et al. Mixed-forest species establishment in a monodominant forest in central Africa: Implications for tropical forest invasibility. PLoS One. 2014;9(5). doi: 10.1371/journal.pone.0097585 PMC4028239 24844914

[24] Lokonda M, Freycon V, Gourlet-Fleury S, Kombele F. Are soils under monodominant *Gilbertiodendron dewevrei* and under adjacent mixed forests similar? A case study in the Democratic Republic of Congo. Journal of Tropical Ecology. 2018;34(3):176–185. doi: 10.1017/S0266467418000135

[25] Newbery D, Van Der Burgt X, Worbes M, Chuyong G. Transient dominance in a central African rain forest. Ecological Monographs. 2013;83(3):339–382. doi: 10.1890/12-1699.1

[26] Umunay P, Gregoire T, Ashton M. Estimating biomass and carbon for *Gilbertiodendron dewevrei* (De Wild) Leonard, a dominant canopy tree of African tropical rainforest: Implications for policies on carbon sequestration. Forest Ecology and Management. 2017;404:31–44. doi: 10.1016/j.foreco.2017.08.020

[27] Thomas, SC, Makana, J-R, Hart, TB, Hart JA, Condit, R, Ewango, CEN Deterministic changes in species composition in primary forest in the Eastern Congo basin [abstract, unpublished]. Ecological Society of America 90th Annual Meeting. 2005, Montreal, Canada.

[28] McGuire KL Common ectomycorrhizal networks may maintain monodominance in a tropical rain forest. Ecology.2007;88(3):567–574. doi: 10.1890/05-1173 17503583

[29] Makana JR, Hart T, Liengola I, Ewango C, Hart J, Condit R. Ituri forest dynamics plots, Democratic Republic of Congo. In: Tropical Forest Diversity and Dynamism: Findings from a Large-Scale Plot Network. Chicago, USA: University of Chicago Press; 2004. p. 492–505.

[30] Baddeley A, Rubak E, Turner R. Spatial Point Patterns: Methodology and Applications with R. London, UK: Chapman and Hall/CRC Press; 2015. Available from: http://www.crcpress.com/Spatial-Point-Patterns-Methodology-and-Applications-with-R/Baddeley-Rubak-Turner/9781482210200/.

[31] Baddeley A, Turner R. spatstat: An R package for analyzing spatial point patterns. Journal of Statistical Software. 2005;12(6):1–42. doi: 10.18637/jss.v012.i06

[32] Gregoire TG, Valentine HT. Sampling Strategies for Natural Resources and the Environment. Chapman and Hall/CRC; 2007.

[33] Hijmans RJ, Van Etten J, Cheng J, Mattiuzzi M, Sumner M, Greenberg JA, et al. Package ‘raster’. Comprehensive R Archive Network (CRAN); 2019. Available from: https://cran.r-project.org/web/packages/raster/vignettes/Raster.pdf.

[34] Tomlin CD. GIS and Cartographic Modeling. Esri Press; 2012.

[35] Keitt TH, Urban DL, Milne BT. Detecting critical scales in fragmented landscapes. Conservation Ecology. 1997;1(1). doi: 10.5751/ES-00015-010104

[36] McGarigal K. FRAGSTATS help. University of Massachusetts: Amherst MA, USA; 2015. Available from: https://www.umass.edu/landeco/research/fragstats/documents/fragstats.help.4.2.pdf.

[37] Bivand RS, Rundel C, Pebesma EJ, Stuetz R, Hufthammer KO, Giraudoux P, et al. Package ‘rgeos’. Comprehensive R Archive Network (CRAN); 2019. Available from: https://cran.r-project.org/web/packages/rgeos/rgeos.pdf.

[38] Van der Burgt XM, Mackinder BA, Wieringa JJ, de la Estrella M. The *Gilbertiodendron ogoouense* species complex (*Leguminosae: Caesalpinioideae*), Central Africa. Kew bulletin. 2015;70(2):29. doi: 10.1007/s12225-015-9579-4

[39] Van der Burgt XM, Eyakwe M, Motoh J. Gilbertiodendron newberyi (*Leguminosae:Caesalpinioideae*), a new tree species from Korup National Park, Cameroon. Kew Bulletin. 2012;67(1):51–57. doi: 10.1007/s12225-012-9345-9

[40] Van Der Burgt XM. Explosive seed dispersal of the rainforest tree *Tetraberlinia moreliana* (*Leguminosae–Caesalpinioideae*) in Gabon. Journal of Tropical Ecology. 1997;13(1):145–151. doi: 10.1017/S0266467400010336

[41] Pinheiro JC, Bates D. Mixed-Effects Models in S and S-PLUS. New York, NY: Springer-Verlag; 2009.

[42] Pinheiro J, Bates D, DebRoy S, authors E, Heisterkamp S, Van Willigen B, et al. Package ‘nlme’. Central R Area Network; 2019. Available from: https://cran.r-project.org/web/packages/nlme/nlme.pdf.

[43] Efron B. Better bootstrap confidence intervals. Journal of the American Statistical Association.1987;82(397):171–185. doi: 10.2307/2289153

[44] Jones MC. Simple boundary correction for kernel density estimation. Statistics and Computing. 1993;3(3):135–146. doi: 10.1007/BF00147776

[45] Diggle PJ. Statistical Analysis of Spatial and Spatio-Temporal Point Patterns.CRC; 2014.

[46] Marshall RC. The Physiology and Vegetation of Trinidad and Tobago. vol. 17. Oxford Forestry Memoirs; 1939.

[47] Marshall RC. Silviculture of the Trees of Trinidad and Tobago. Oxford University Press; 1939.

[48] Gunther A. The Distribution and Status of Mora Forest (*Mora excelsa*) in the Ortoire Basin, Trinidad, BWI. Empire Forestry Journal. 1942;21(2):123–127.

[49] Oatham MP, Jodhan D. Is Mora taking over? Testing the limits to the invasive ability of *Mora excelsa* Benth. A pilot study. Living World, Journal of the Trinidad and Tobago Field Naturalists’ Club. 2002.

[50] Eggeling W. Observations on the ecology of the Budongo rain forest, Uganda. The Journal of Ecology. 1947; p. 20–87. doi: 10.2307/2256760

[51] Woolley LP, Henkel TW, Sillett SC. Reiteration in the monodominant tropical tree *Dicymbe corymbosa* (*Caesalpiniaceae*) and its potential adaptive significance. Biotropica. 2008;40(1):32–43.

[52] Charles-dominique P, Chave J, Dubois MA, De Granville JJ, Riera B, Vezzoli C. Colonization front of the understorey palm *Astrocaryum sciophilum* in a pristine rain forest of French Guiana. Global Ecology and Biogeography. 2003;12(3):237–248. doi: 10.1046/j.1466-822X.2003.00020.x

[53] Favier C, De Namur C, Dubois MA. Forest progression modes in littoral Congo, central Atlantic Africa. Journal of Biogeography. 2004;31(9):1445–1461. doi: 10.1111/j.1365-2699.2004.01094.x

[54] Archer S, Scifres C, Bassham C, Maggio R. Autogenic succession in a subtropical savanna: Conversion of grassland to thorn woodland. Ecological Monographs. 1988;58(2):111–127. doi: 10.2307/1942463

[55] Gandhi A, Levin S, Orszag S. Nucleation and relaxation from meta-stability in spatial ecological models. Journal of Theoretical Biology 1999;200(2):121–146. doi: 10.1006/jtbi.1999.0978 10504281

[56] Hopkins B, Jenkin R. Vegetation of the Olokemeji Forest Reserve, Nigeria: I. General features of the reserve and the research sites. The Journal of Ecology. 1962; 50(3):559–598. doi: 10.2307/2257471

[57] Whittaker R, Gilbert L, Connell J. Analysis of two-phase pattern in a mesquite grassland, Texas. The Journal of Ecology. 1979;67(3):935–952. doi: 10.2307/2259222

[58] Archer S. Development and stability of grass/woody mosaics in a subtropical savanna parkland, Texas, USA. Journal of Biogeography. 1990;17:453–462. doi: 10.2307/2845377

[59] Guillet B, Achoundong G, Youta Happi J, Beyala VKK, Bonvallot J, Riera B, et al. Agreement between floristic and soil organic carbon isotope (^13^C/^12^C, ^14^C) indicators of forest invasion of savannas during the last century in Cameroon. Journal of Tropical Ecology. 2001;17(6):809–832. doi: 10.1017/S0266467401001614

[60] Puyravaud JP, Dufour C, Aravajy S. Rain forest expansion mediated by successional processes in vegetation thickets in the Western Ghats of India. Journal of Biogeography. 2003;30(7):1067–1080. doi: 10.1046/j.1365-2699.2003.00882.x

[61] Miège J. Observations sur les fluctuations des limites savanes-forêts en basse Côte d’Ivoire. Annals de la Faculè des Lettres et Sciences Humaines, Univesity of Dakar. 1966;19:149–166.

[62] Hopkins B. Successional Processes. In: Tropical Savannas. New York, NY: Elsevier Scientific Publishing; 1983. p. 605–616.

[63] Schwartz D, Lanfranchi R, Mariotti A. Origine et évolution des savanes intramayombiennes (RP du Congo). I. Apports de la pédologie et de la biogéochimie isotopique (^13^C and ^14^C). Paysages quaternaires de l’Afrique Centrale atlantique ORSTOM, Paris. 1990; p. 314–325.

[64] Schwartz D, De Foresta H, Mariotti A, Balesdent J, Massimba J, Girardin C. Present dynamics of the savanna-forest boundary in the Congolese Mayombe: A pedological, botanical and isotopic (^13^C and ^14^C) study. Oecologia. 1996;106(4):516–524. doi: 10.1007/BF00329710 28307452

[65] Delègue MA, Fuhr M, Schwartz D, Mariotti A, Nasi R. Recent origin of a large part of the forest cover in the Gabon coastal area based on stable carbon isotope data. Oecologia. 2001;129(1):106–113. doi: 10.1007/s004420100696 28547057

[66] Fabing A. Bilan Spatial et Structurel de l’Antagonisme ‘Pression Anthropique/Dynamique Forestiere Naturelle’ en Zone de Forte Croissance Urbaine. Unpublished Thesis, University Louis Pasteur, Strasbourg. 2000.

[67] Youta Happi J. Arbres contre graminées: La lente invasion de la savane par la forêt au Centre-Cameroun. Unpublished thesis, Universite Paris IV. 1998.

[68] Cuni-Sanchez A, White LJ, Calders K, Jeffery KJ, Abernethy K, Burt A, et al. African savanna-forest boundary dynamics: A 20-year study. PLoS One. 2016;11(6):e0156934. doi: 10.1371/journal.pone.0156934 27336632 PMC4919100

[69] Gandhi A, Levin S, Orszag S. “Critical slowing down” in time-to-extinction: An example of critical phenomena in ecology. Journal of Theoretical Biology 1998;192(3):363–376. doi: 10.1006/jtbi.1998.0660 9650292

[70] Lozon JD, MacIsaac HJ. Biological invasions: Are they dependent on disturbance? Environmental Reviews. 1997;5(2):131–144.

[71] Yamamoto SI. Forest gap dynamics and tree regeneration. Journal of Forest Research. 2000;5(4):223–229. doi: 10.1007/BF02767114

[72] Tanentzap AJ, Bazely DR. Propagule pressure and resource availability determine plant community invasibility in a temperate forest understorey. Oikos. 2009;118(2):300–308. doi: 10.1111/j.1600-0706.2008.17069.x

[73] Sanford NL, Harrington RA, Fownes JH. Survival and growth of native and alien woody seedlings in open and understory environments. Forest Ecology and Management. 2003;183(1-3):377–385. doi: 10.1016/S0378-1127(03)00141-5

