# Supplementary figures and images for: The spatial propagation and increasing dominance of Gilbertiodendron dewevrei (Fabaceae) in the eastern Congo basin
Source: PLoS One. 2023 Feb 7;18(2):e0275519. doi: 10.1371/journal.pone.0275519 (PMC9904485; doi:10.1371/journal.pone.0275519)

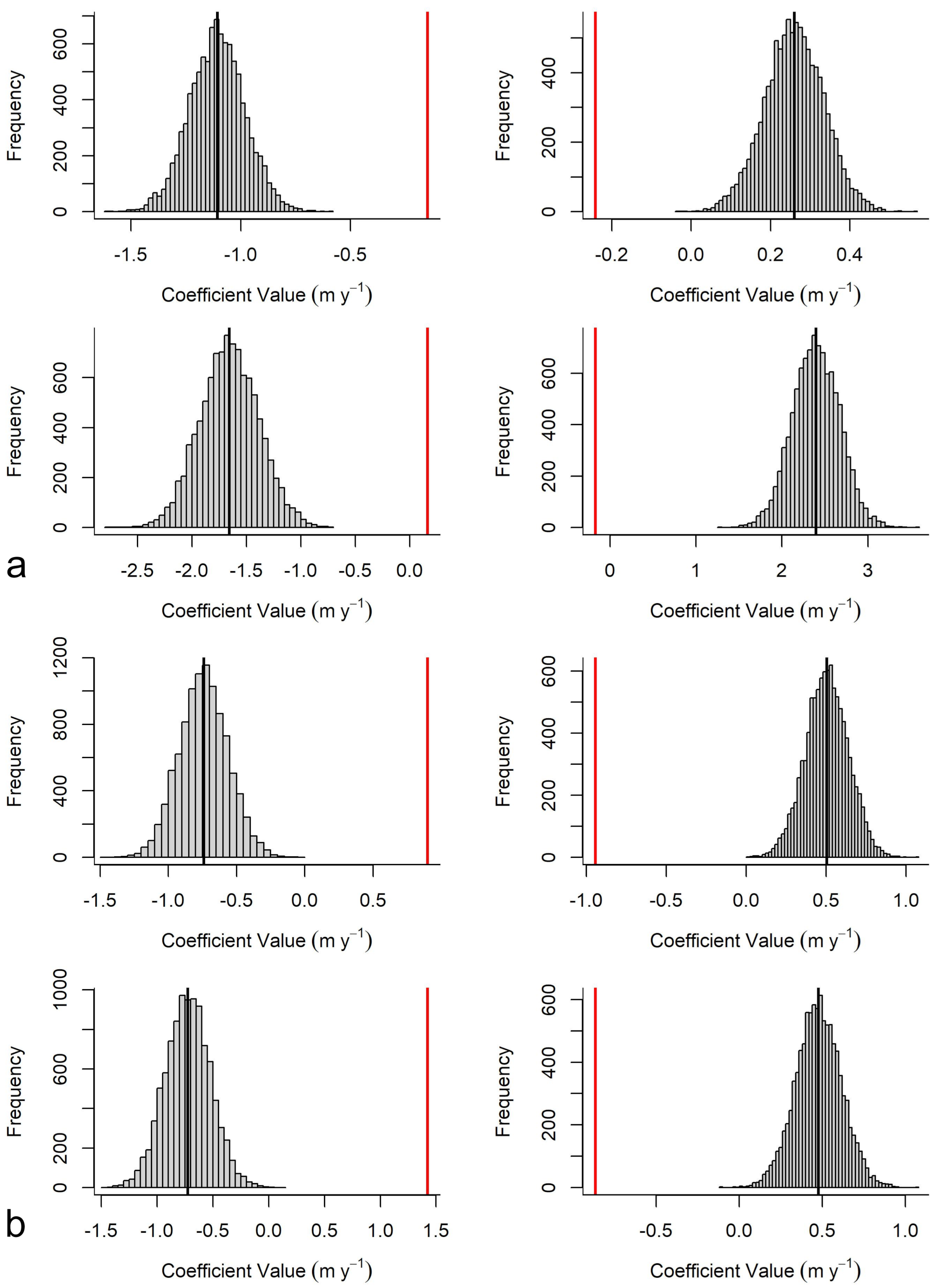

Supplement: S1 Fig — Graphical results of permutation tests (n = 10, 000 iterations) of isoline movement models for site edoro1, patch 1 (a) and patch 2 (b). Histograms capture the null distribution of movement coefficients (m y−1) for each directional quadrant: N, S, W, E (clockwise from upper left). The black vertical line is the mean of the distribution, and the red vertical line captures the results of our observed algorithmic pairing of isoline vertices between the third and first censuses. All results highly significant at α=0.05. (TIF) [file pone.0275519.s001.tif]

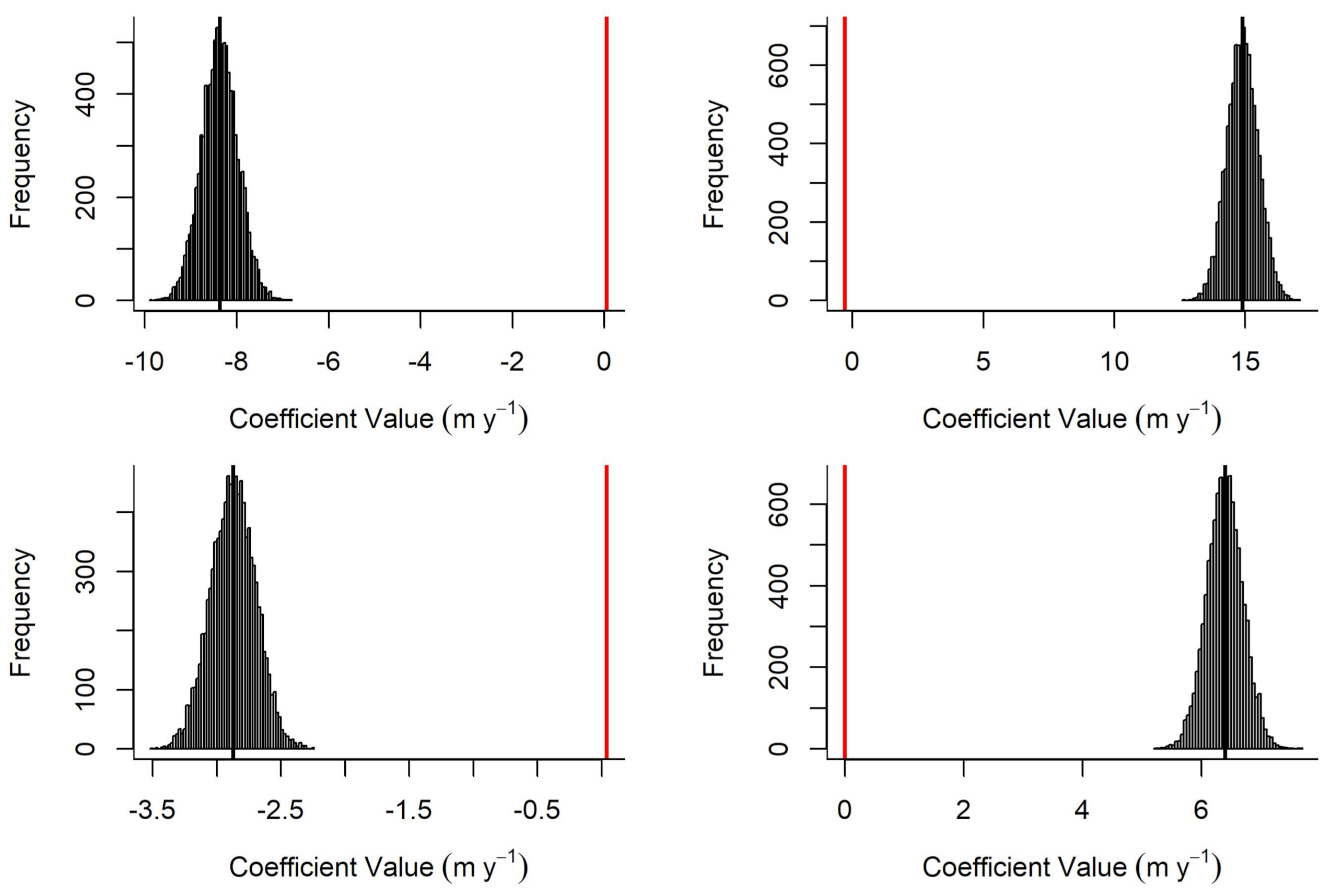

Supplement: S2 Fig — Graphical results of permutation tests (n = 10, 000 iterations) of isoline movement models for site lenda1, patch 1. Histograms capture the null distribution of movement coefficients (m y−1) for each directional quadrant: N, S, W, E (clockwise from upper left). The black vertical line is the mean of the distribution, and the red vertical line captures the results of our observed algorithmic pairing of isoline vertices between the third and first censuses. All results highly significant at α=0.05. (TIF) [file pone.0275519.s002.tif]

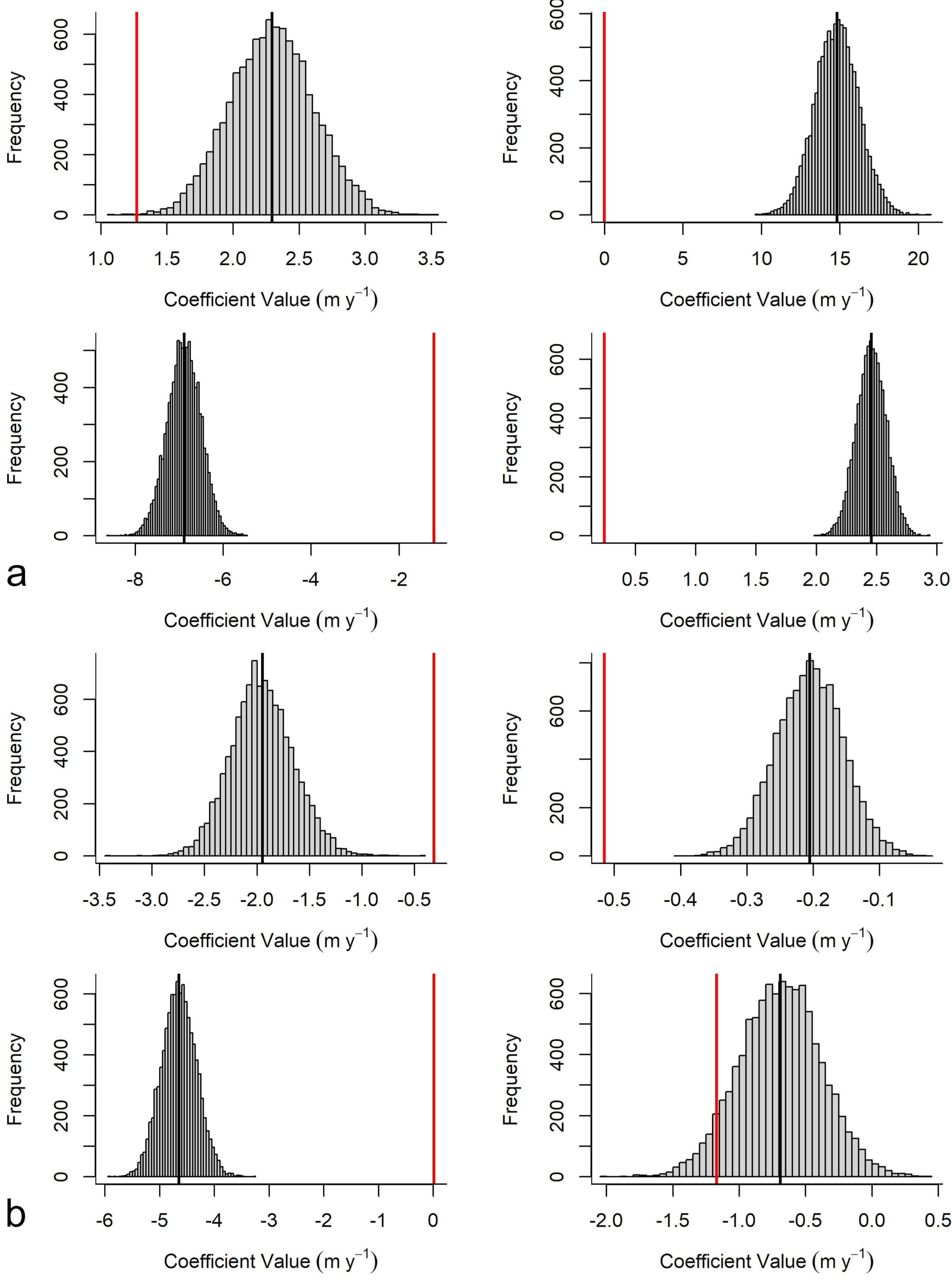

Supplement: S3 Fig — Graphical results of permutation tests (n = 10, 000 iterations) of isoline movement models for site lenda2, patch 1 (a) and patch 2 (b). Histograms capture the null distribution of movement coefficients (m y−1) for each directional quadrant: N, S, W, E (clockwise from upper left). The black vertical line is the mean of the distribution, and the red vertical line captures the results of our observed algorithmic pairing of isoline vertices between the third and first censuses. All results highly significant at α=0.05, except patch 2 W (p = 0.06). (TIF) [file pone.0275519.s003.tif]
